# RNA-Seq-based transcriptomic and metabolomic analysis reveal stress responses and programmed cell death induced by acetic acid in *Saccharomyces cerevisiae*

**DOI:** 10.1038/srep42659

**Published:** 2017-02-17

**Authors:** Yachen Dong, Jingjin Hu, Linlin Fan, Qihe Chen

**Affiliations:** 1 Department of Food Science and Nutrition, Key Laboratory for Food Microbial Technology of Zhejiang Province, Zhejiang University, 866 Yuhangtang Road, Hangzhou 310058, China

## Abstract

As a typical harmful inhibitor in cellulosic hydrolyzates, acetic acid not only hinders bioethanol production, but also induces cell death in *Saccharomyces cerevisiae*. Herein, we conducted both transcriptomic and metabolomic analyses to investigate the global responses under acetic acid stress at different stages. There were 295 up-regulated and 427 down-regulated genes identified at more than two time points during acetic acid treatment (150 mM, pH 3.0). These differentially expressed genes (DEGs) were mainly involved in intracellular homeostasis, central metabolic pathway, transcription regulation, protein folding and stabilization, ubiquitin-dependent protein catabolic process, vesicle-mediated transport, protein synthesis, MAPK signaling pathways, cell cycle, programmed cell death, etc. The interaction network of all identified DEGs was constructed to speculate the potential regulatory genes and dominant pathways in response to acetic acid. The transcriptional changes were confirmed by metabolic profiles and phenotypic analysis. Acetic acid resulted in severe acidification in both cytosol and mitochondria, which was different from the effect of extracellular pH. Additionally, the imbalance of intracellular acetylation was shown to aggravate cell death under this stress. Overall, this work provides a novel and comprehensive understanding of stress responses and programmed cell death induced by acetic acid in yeast.

Nowadays, there is a great need for renewable biofuels to reduce reliance on fossil fuels^1^. Cellulosic ethanol is an ideal clean biofuel, which can be produced via saccharification and fermentation of acidic hydrolysates from the lignocellulosic biomass^2^. *Saccharomyces cerevisiae* is considered as a worthy biocatalyst for ethanol conversion owing to its high productivity and robust performance^3^. However, there are some inhibitors in lignocellulosic hydrolysates, which impair yeast growth and bioethanol yield. Specifically, acetic acid is a predominant inhibitor with the high concentration typically ranging from 1 to 15 g/L in the hydrolysates^4^. The productivity and yield of cellulosic ethanol have been economically hampered by the toxicity of acetic acid in the acidic hydrolysates. It is difficult to comprehensively understand the inhibitory effect of complex inhibitors in the hydrolysates. Nevertheless, we can take the first step to investigate the toxic effects of acetic acid on yeast growth under acidic conditions.

At an extracellular pH below 4.76 (pK_a_), the undissociated acetic acid enters yeast cells primarily by passive diffusion, and dissociates into acetate and protons in neutral cytoplasm^5^. The protons can be pumped out of cells by ATPase Pma1p under low concentration of acetic acid^6^. Meanwhile, acetate can be metabolized to acetyl-CoA by Acs1p (peroxisomal) or Acs2p (cytosolic), then oxidized in the tricarboxylic acid (TCA) cycle, consumed in the glyoxylate shunt, or used for the synthesis of macromolecules by gluconeogenesis^7^. These suggest a potential connection between acetic acid and the acetyl-CoA pool, which is essential for intermediary metabolism, histone acetylation, and transcriptional regulation^8^,^9^. However, the metabolism of acetic acid is generally subjected to glucose repression in *S. cerevisiae*^5^. Little is known about the impact of acetate increasingly accumulating in yeast cells upon acetic acid stress.

High levels of acetic acid can inhibit yeast cell growth, but a low external pH causes comparable growth inhibition with a lower acetic acid concentration^5^, and even induces programmed cell death (PCD) with typical phenotypes of morphology and physiology^10^. The mitochondria-dependent death process is also activated during acetic acid treatment^11^. Different approaches, such as phenotypic screening of the mutant collections, proteomics and metabolomics analysis, are applied to identify the genes, proteins, and metabolic pathways in response to acetic acid stress^12^,^13^,^14^. Moreover, Lee *et al*. have compared six transciptome datasets for genes regulated by acetic acid in yeast under various conditions, including different strains, extracellular pH, and acetic acid concentrations, but all of the data hardly agree well with each other^15^. Therein, five studies employed microarray analysis to evaluate the transcriptional changes in acetic acid treated cells^16^,^17^,^18^,^19^,^20^. With the advent of next-generation sequencing, high-throughput mRNA sequencing (RNA-Seq) has become an attractive alternative for transcriptomic analysis, which can uncover novel transcriptional-related events and quantify the expression genome-wide in a single assay with high resolution, better dynamic range of detection, and lower technical variation^21^,^22^.

The toxic effects of acetic acid in lignocellulosic hydrolysates mainly derive from three aspects: high concentration, low pH, and nitrogen limitation. Nitrogen starvation can also induce bulk degradation in yeast^23^. Therefore, we performed the experiments with 150 mM acetic acid (pH 3.0) for different times in a synthetic complete (SC) medium supplemented with only auxotrophic amino acids and nucleotides. Both transcriptomic and metabolomic analyses were used to investigate the global responses of yeast cells under acetic acid stress and identify the regulatory mechanisms for rerouting metabolic fluxes. Cell viability and mitochondrial degradation were firstly measured under different external pH or acetic acid concentrations. Cytosolic pH (pH_cyt_) and mitochondrial pH (pH_mit_) were *in situ* monitored using a pH-sensitive ratiometric pHluorin in treated and untreated cells^24^. Furthermore, the phenotypic properties of yeast cells were confirmed by PCD assay, scanning and transmission electron microscopies. The imbalance of histone acetylation was also analyzed to evaluate the impact on cell death under acetic acid stress. These findings suggest new insights into how yeast cells respond to acetic acid stress, and contribute to the exploration of the engineered *S. cerevisiae* strains with a high inhibitor tolerance for bioethanol production.

## Results

### Acetic acid induces cell death and mitochondrial degradation differing from the effect of low extracellular pH

Acetic acid triggers PCD in yeast cells with a typical feature of mitochondrial degradation^11^, and the weak acid toxicity is aggravated in the medium at a lower pH^5^. To distinguish the effects of extracellular pH and acetic acid on cell death, we respectively compared cell viability and mitochondrial degradation in *S. cerevisiae* W303–1B. In this work, cell viability was measured by colony forming unit (CFU) counts. Mitochondrial matrix-targeted green fluorescent protein (GFP) has been proved to be an effective method to detect mitochondrial degradation by flow cytometry^11^, thus we analyzed mitochondrial degradation in W303 strain transformed with pYX232-mtGFP under the control of TPI promoter^25^ by measuring the percentage of cells that lost mtGFP fluorescence.

**Figure 1:**
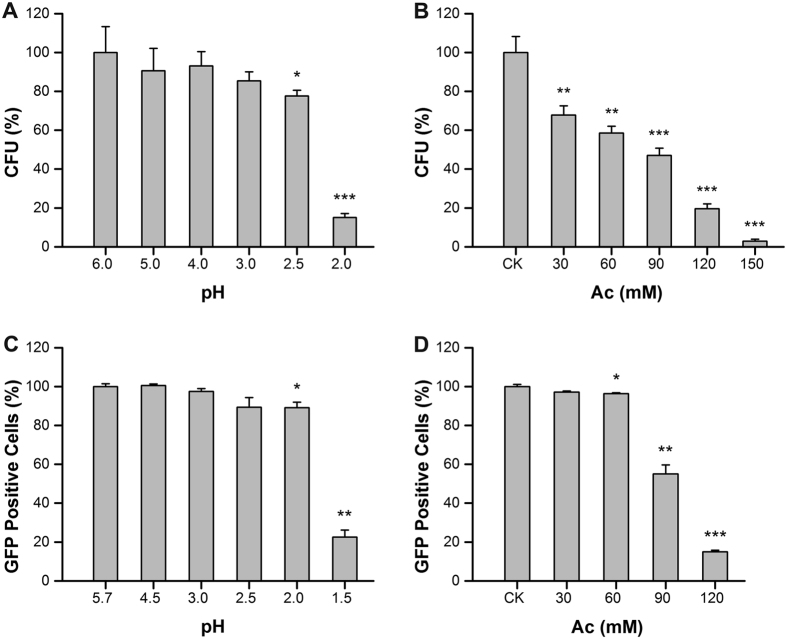
Cell viability and mitochondrial degradation of *S. cerevisiae* under different conditions. (**A**,**B**) CFU assay under different external pH or acetic acid concentrations, respectively. (**C**,**D**) Loss of mitochondrial GFP-positive cells under different external pH or acetic acid concentrations, respectively. Values are mean ± S.D. (n = 3). *P < 0.05, **P < 0.01, ***P < 0.001, two-tailed *t* test.

We first compared the cell viability and mitochondrial degradation in yeast cells under different culture pH levels without acetic acid treatment. The culture samples were collected and spotted on the solid YPD medium at 28 °C for 2 d. We observed no significant difference (*P* > 0.05, two-tailed Student’s *t* test) in cell survival with the culture pH from 6.0 to 3.0 (adjusted with 1 M HCl), but a steep decline in cell viability at extracellular pH levels of 2.0–2.5 (Fig. 1A). In contrast, the cell viability decreased significantly under acetic acid treatment (≥30 mM) at the same extracellular pH 3.0 (Fig. 1B). Likewise, there was no delay in GFP disappearance in the media without acetic acid at a pH range of 3.0–5.7, but a decrease in GFP-positive cells when the acetic acid concentration was more than 60 mM at pH 3.0 (Fig. 1C,D). A sharp increase in mitochondrial degradation was also observed when the pH fell to 1.5 in untreated cells (Fig. 1C). Thus, it can be seen that the change of extracellular pH from 6.0 to 3.0 without acetic acid treatment has no significant impact on cell death in *S. cerevisiae (P* > 0.05, two-tailed *t* test), but acetic acid induces cell death with the concentration above 30 mM when the culture pH remains at 3.0. In order to explore the truth behind it, we chose a severe condition (150 mM acetic acid, pH 3.0) for further study.

### Acetic acid results in typical phenotypes of programmed cell death

**Figure 2:**
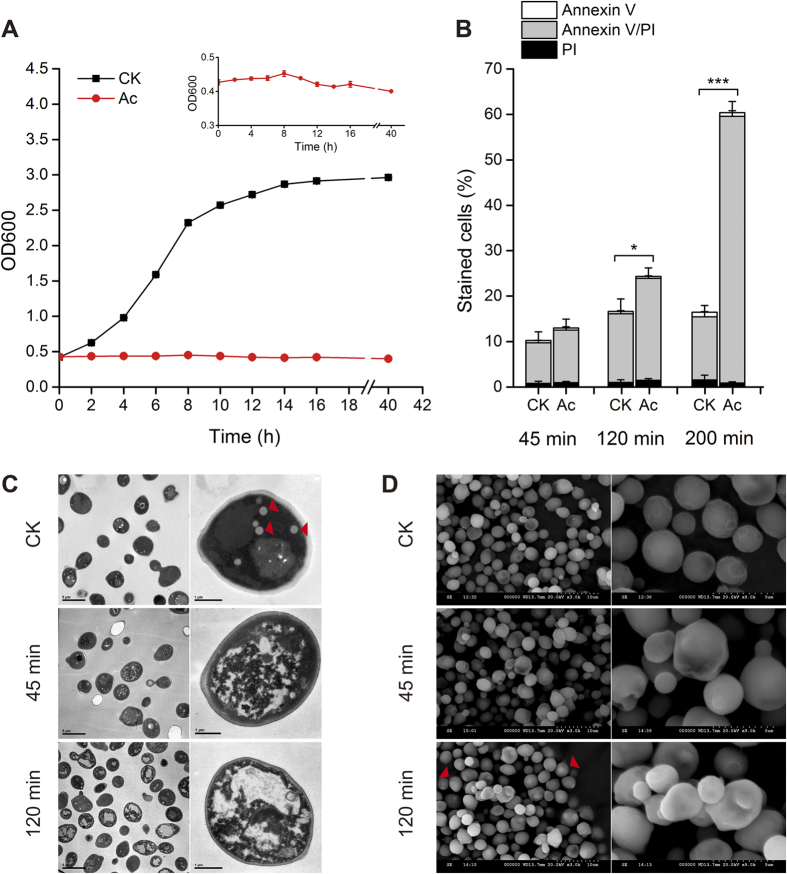
Comparison of phenotypic properties in yeast cells with and without acetic acid treatment at different times. (**A**) Growth curves of treated and untreated cells, with an enlarged image of treated cells in the insert. (**B**) PCD assay. Values are mean ± S.D. (n = 3). *P < 0.05, ***P < 0.001, two-tailed *t* test. (**C**) TEM observation. Lipid droplets are indicated by arrows. (**D**) SEM observation. Disrupted cells are indicated by arrows.

Though acetic acid-induced PCD has been widely reported in yeast strains^5^,^10^,^11^, there is still a need for a better understanding of the cascading events. Accordingly, the phenotypic properties of yeast cells were compared in SC1 medium (0.67% (w/v) yeast nitrogen base without amino acids (YNB), 2% (w/v) D-glucose, 0.004% (w/v) histidine, 0.008% (w/v) leucine, 0.004% (w/v) tryptophan, 0.004% (w/v) adenine and 0.004% (w/v) uracil, pH 3.0) with and without 150 mM acetic acid. In Fig. 2A, we observed a typical S-shaped growth curve in the control group (CK). In the presence of acetic acid, yeast growth was seriously inhibited, although there was a slight increase during the first 8 h (Fig. 2A). Since FITC-conjugated Annexin V and propidium iodide (PI) have been employed to monitor phosphatidylserine externalization and loss of membrane integrity, Annexin V/PI co-staining was performed to distinguish early apoptosis (Annexin V+, PI−), late apoptosis (Annexin V+, PI+), and primary necrosis (Annexin V−, PI+)^26^. During acetic acid treatment (150 mM, pH 3.0), W303-1B cells mainly showed an increase in late apoptotic populations from 45 min to 200 min (Fig. 2B). In contrast, there were no significant changes (*P* > 0.05, two-tailed *t* test) in cell death during the treatment without acetic acid.

Next, transmission electron microscopy (TEM) analysis revealed the intracellular structures were greatly changed in many yeast cells treated with acetic acid (Fig. 2C). The untreated cells exhibited intact structures of organelles and cell nucleus, excessive accumulation of lipid droplets (LD, red arrows), with a thick and transparent cell wall. After acetic acid treatment, vacuolation of the cytoplasm was increasingly observed in yeast cells, intracellular organelles and cell nucleus were rapidly disintegrated or collapsed, the accumulation of LD was abolished, and the cell wall became thinner with a darker color. There was extensive chromatin condensation along with DNA fragmentation in cells treated with acetic acid. In addition, the surface morphology in yeast cells with and without acetic acid treatment was compared using scanning electron microscopy (SEM). As shown in Fig. 2D, the rough surface in untreated cells became much smoother, and some cells were ruptured (red arrows) after incubation with 150 mM acetic acid (pH 3.0), indicating the yeast cells became vulnerable to this weak acid.

### Intracellular acidification is induced upon acetic acid stress in cytosol and mitochondrial matrix

Intracellular pH (pH_i_) is tightly linked to cellular signal regulation in yeast^27^. It has been viewed as an effective method for *in situ* pH_i_ measurements in *S. cerevisiae* by expressing the pH-sensitive GFP ratiometric pHluorin^24^,^28^. pH_cyt_ and pH_mit_ can be determined by transforming the multicopy plasmids pYES2-ACT1-pHluorin and pYES2-ACT1-mtpHluorin^24^. The latter was fused with a mitochondrial targeting signal of 69 amino acids in upstream of F0-ATPase subunit 9 in *Neurospora crassa*^24^, which made it possible to determine the pH value in the mitochondrial matrix. The *in situ* calibration curves for pH_cyt_ and pH_mit_ measurements are shown in Fig. S1.

**Figure 3:**
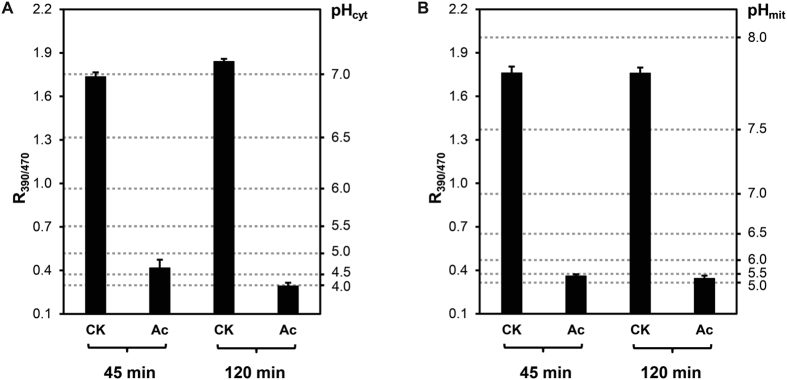
Change of pH_i_ in yeast cells under acetic acid stress. (**A**) *In situ* pH_cyt_ of treated (Ac) and untreated (CK) cells at different times. (**B**) *In situ* pH_mit_ of yeast cells at different times. Values are mean ± S.D. (n ≥ 3).

In untreated cells, the pH_cyt_ was 6.98 ± 0.03 at 45 min, and slightly increased to 7.08 ± 0.02 at 120 min, although the external pH in medium was 3.0 (Fig. 3A). These indicated that yeast cells could maintain a relatively neutral pH in cytosol under the acidic condition. In contrast, the pH_cyt_ decreased dramatically in yeast cells after acetic acid treatment for 45 min and 120 min, which were 4.66 ± 0.22 and 3.94 ± 0.22, respectively (Fig. 3A). Simultaneously, the pH_mit_ in cells treated with acetic acid were respectively 5.38 ± 0.09 (45 min) and 5.22 ± 0.17 (120 min), while the untreated cells showed a pH_mit_ around 7.83 in different culture times (Fig. 3B). These data show that yeast cells can also maintain a mitochondrial pH with a slight alkalinity in acidic medium (pH 3.0), but acetic acid treatment results in severe intracellular acidification in the cytosol and mitochondrial matrix. Although yeast cells have an ability to restore pH_i_ when the concentration of acetic acid is below 60 mM^6^, the pH_i_ recovery was greatly suppressed at the concentration of 150 mM acetic acid. In view of the above data at same conditions, the severe acidification probably leads to intracellular damage and degradation, thus inducing programmed cell death.

### Global transcriptional changes are stimulated by acetic acid stress

To investigate the stress responses under acetic acid stress, we performed transcriptome analysis in *S. cerevisiae* cells at different treatment times. Two high-quality mRNA samples were selected for RNA-Seq analysis from three independent experiments for each condition. All the raw data have been registered at the Sequence Read Archive in NCBI under accession number SRP075510. Further details of the RNA-Seq data are provided in Table S1. Q20 and Q30 of all clean reads were respectively more than 99.2% and 95.8%, thus the RNA-Seq data were of high quality for transcriptome analysis.

Next, RNA-Seq data were validated by quantitative real-time polymerase chain reaction (qPCR) for analysis of mRNA expression. Fifteen genes with distinct fold changes in different biological pathways were selected for verification (Fig. S2A). As shown in Fig. S2B, a high correlation was observed between the RNA-Seq and qPCR data (*R*^2^ = 0.96), demonstrating the validity of RNA-Seq data for genes with various transcript abundances.

**Figure 4:**
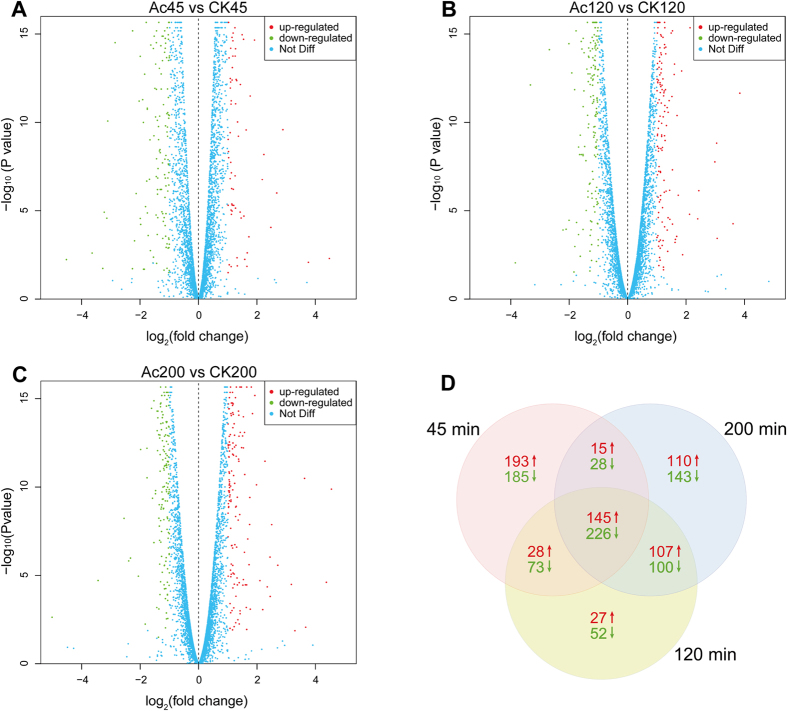
Comparison of gene expression between yeast cells with and without acetic acid at different times. (**A**–**C**) Scatterplot matrix comparison of gene expression in treated (Ac) and untreated (CK) cells at 45 min, 120 min and 200 min, respectively. (**D**) Venn diagram of DEGs at the three time points.

The differentially expressed genes (DEGs) were identified to show significant change in transcriptional expression with more than 2-fold change (*q*-value < 0.05). There were respectively 893, 758, and 874 DEGs at 45 min, 120 min, and 200 min after treatment of 150 mM acetic acid (pH 3.0) (Fig. 4). Comparing to the control groups (CK), 381, 307, and 377 genes were up-regulated in acetic acid treated groups (Ac) at three time points, while 512, 451, and 497 genes were down-regulated (Fig. 4D). The large number of DEGs suggests that acetic acid has global effects on stress responses and PCD in *S. cerevisiae*, and more DEGs were down-regulated than up-regulated in general.

More than 94.7% of DEGs at different times are annotated with GO terms. The overrepresented functional categories are presented in Fig. S3. Among all three GO functional categories, the proportions of DEGs in corresponding subcategories at three time points were mainly consistent with each other. The most dominant subcategories were involved in cellular process (88.9% DEGs at 45 min, 89.2% at 120 min, 88.8% at 200 min), metabolic process (71.6%, 71.0%, 73.5%), and biosynthetic process (35.2%, 39.3%, 41.6%) in biological process; organelle (71.1%, 70.8%, 73.7%), nucleus (37.1%, 33.0%, 32.7%), and membrane (35.5%, 37.3%, 33.9%) in cellular component; binding (49.2%, 45.9%, 47.8%), catalytic activity (42.6%, 42.2%, 38.4%), and ion binding (29.5%, 26.4%, 26.5%) in molecular function.

All DEGs were further mapped to terms in the Kyoto Encyclopedia of Genes and Genomes (KEGG, http://www.kegg.jp/) database. There were 303, 321, and 418 DEGs at different times matched to KEGG pathways. As illustrated in Fig. S4, ‘metabolic pathways’ was the largest category, containing 16.9%, 17.3%, and 15.2% DEGs at three time points (45 min, 120 min, and 200 min). Among them, there were many DEGs (3.9%, 5.9%, and 3.9%) involved in ‘biosynthesis of amino acids’. It was indicated that some coordinated changes appeared in global metabolic pathways for yeast cells upon acetic acid stress over time, whereas the proportion of ribosome protein-coding genes in DEGs was rapidly increased from 2.7% (45 min) to 13.3% (200 min), suggesting that the expression of ribosomal genes was greatly changed over time during acetic acid treatment. Herein, the cross-DEGs at more than two time points were first selected for investigating the differential expression of core biological functions and pathways under acetic acid stress (Table S2).

### Cross-DEGs reveal core biological functions and pathways in response to acetic acid stress

**Figure 5:**
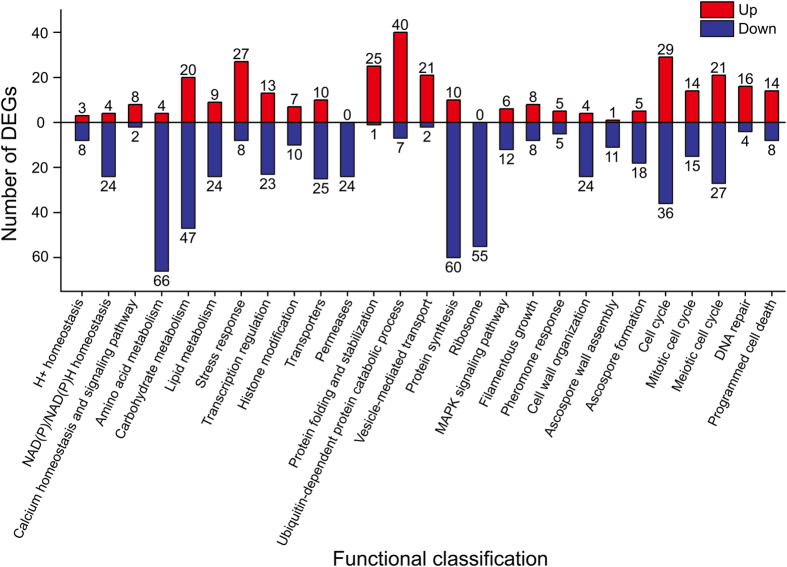
Functional classifications of the cross-DEGs upon acetic acid stress at more than two time points. The numbers of up-regulated and down-regulated DEGs are highlighted in red and blue, respectively.

There are four renowned databases, namely KEGG, UniProt (The Universal Protein Resource, http://www.uniprot.org/), SGD (Saccharomyces Genome Database, http://www.yeastgenome.org/), and MIPS (Munich Information Center for Protein Sequences, http://mips.helmholtz-muenchen.de/funcatDB), which are professional for the analyses of gene functions and metabolic pathways in *S. cerevisiae*. However, there are still some differences in functional annotations and classifications for yeast genes owing to different algorithms and update issues. Herein, 722 cross-DEGs (295 up-regulated and 427 down-regulated, Table S3) at more than two time points were systematically categorized using these four databases, and confirmed by literature retrieval. All of the DEGs were annotated according to the SGD and UniProt databases. In this section, the common DEGs at three time points were emphasized in bold, with up (↑) and down arrow (↓) respectively indicating up- and down-regulation. The well-represented biological pathways were involved in cellular homeostasis, central metabolic pathway, stress response, transcription regulation and histone modification, cellular uptake and transport, ubiquitination process, protein synthesis, MAPK signaling pathway, cell cycle and DNA repair, and programmed cell death (Fig. 5).

Intracellular ionic and redox homeostases are essential for the maintenance of cell survival and metabolism. Upon acetic acid stress, 8 genes (***GDT1**, MIR1, **NCE103**, **NHA1**, **OPT1**, **PMA1**, **PMA2***, and ***VMA1***) sustaining pH homeostasis in cytosol were significantly down-regulated at different times. Likewise, 24 DEGs in NAD(P)/NAD(P)H homeostasis were greatly inhibited by acetic acid, which were mainly correlated to NAD+ synthesis (*SDT1, **BNA2**, BNA3, **BNA4***, and ***FUN26***), and redox transformation from NAD(P) to NAD(P)H (*ADH1, **ALD3**, ALD5, ALD6, **GDH1**, GDH2, GDH3, **GLT1**, GND2, IDP1, **IDP2**, **ILV5**, LYS9, **MET13**, **MTD1***, and ***YEF1***). These would aggravate intracellular acidification induced by acetic acid in *S. cerevisiae*. Moreover, the up-regulated DEGs in calcium homeostasis and signaling pathway (***CDC31**, **CMK2**, **LCB5**, **MDM10**, **MMM1**, **PTP2**, **RCN1***, and ***REE1***) likely had an important impact on signal transduction and stress responses in acetic acid treated cells.

Of the global metabolic pathway, 157 DEGs were identified in the central metabolic pathway, including amino acid metabolism (4 up-regulated and 66 down-regulated genes), carbohydrate metabolism (20 up-regulated and 47 down-regulated genes), and lipid metabolism (9 up-regulated and 24 down-regulated genes). Almost all of the DEGs in the biosynthesis of amino acids were down-regulated, and 3 up-regulated genes (***BAT2**, **CHA1**, EHD3*) were involved in the catabolism of amino acid, suggesting that the biosynthesis of amino acids was suppressed by acetic acid treatment. Interestingly, most of the DEGs in downstream of carbohydrate metabolism, which is next to amino acid metabolism, were also negatively influenced, but many upstream DEGs in carbohydrate metabolism were up-regulated in response to acetic acid stress. Specifically, the down-regulated DEGs mainly included glycolysis process (*ADH1, **ALD3**, ALD5, ALD6, CDC19, ENO1, GID8, **GLK1**, HSP31, NDE2, PCK1, PDC1, **PDC6**, PGM1*, and ***YIG1***), pyruvate metabolism (***ACC1**, ALD5, ALD6, CDC19, DAL7, **GLO4**, HSP31, **LYS21**, PCK1*, and *PDC1*), and NAD(P)/NAD(P)H homeostasis (*ADH1, **ALD3**, ALD5, ALD6, GND2, IDP1, **IDP2**, NDE2*, and *YHM2*). Among the up-regulated DEGs, there were 9 genes (***EMI2**, **FLO11**, GLC3, **GSY1**, **HXK1**, IMA1, MAL12, **PRM15***, and ***YPI1***) involved in starch and sucrose metabolism. In addition, lipid metabolism was likely repressed by acetic acid stress, in which three key genes (***FAS1**, **FAS2***, and ***ACC1***) of fatty acid biosynthesis were significantly down-regulated.

Transcription factors are essential for yeast cells in response to environmental stresses. There were 13 DEGs up-regulated by acetic acid stress, but more DEGs (23 genes) were repressed. These might explain why more genes in acetic acid-treated cells were down-regulated rather than up-regulated at three time points, in comparison with the untreated cells. These DEGs in transcription regulation were related to various biological processes, including biosynthesis of amino acids (***LEU3**, LYS14*, and ***MET32***), carbohydrate metabolism (***MIG1**, MIG2*, and ***MIG3***), histone modification (*ADA2, BYE1, **ESA1**, HST3, **SET2***, and ***SGF29***), MAPK signaling pathway (***SMP1***), cell cycle (*BYE1, MSA2, NDT80, **PPH22**, SFH1, SWI5*, and ***YOX1***), ubiquitination process (*RAD6*), DNA repair (***ESA1**, **NHP6A***, and *RAD6*), and stress response (*CIN5*). In histone modification, there were four up-regulated DEGs (***AHC2**, **ESA1**, HPA2*, and ***YAF9***) required for histone acetylation, and three down-regulated DEGs (***HOS1**, HST3*, and ***SET2***) involved in histone deacetylation, suggesting that cellular histone acetylation was enhanced in response to acetic acid. In addition, ***ESA1*** is required for the regulation of autophagy^29^.

Not only was intracellular metabolism greatly changed, but the uptake and transport of various nutrients were seriously inhibited by acetic acid (150 mM, pH 3.0). A number of genes encoding permeases (24 genes) and transporters (25 genes) were down-regulated during acetic acid treatment. Among them, there were 14 amino acid permeases (***AGP1**, **AGP3**, BAP3, **CAN1**, **DIP5**, **GAP1**, **GNP1**, HIP1, **LYP1**, **MMP1**, **MUP1**, **PUT4**, **SAM3***, and *UGA4*), 15 plasma membrane transporters (***ALP1**, **ATO3**, DUR3, **HNM1**, **HXT1**, HXT5, **ITR1**, **NHA1**, **OPT1**, PHO84, **QDR3**, TAT1, TPO3, **YHK8***, and *ZRT2*), 5 mitochondrial inner membrane transporters (***CTP1**, GGC1, **OAC1**, **ODC2***, and ***SFC1***), 3 vacuolar membrane transporters (***CTR2**, **FUN26***, and ***RTC2***) and 2 endoplasmic reticulum transporters (***FLC1***and ***YKE4***). Interestingly, 6 out of 10 up-regulated transporter genes (***HXT6**, **HXT7**, HXT9, HXT10, HXT11*, and ***HXT13***) are encoding genes of hexose transporters, which are consistent with the elevated gene expression levels upstream of carbohydrate metabolism.

Under acetic acid stress, 25 DEGs involved in protein folding and stabilization were induced to suppress protein aggregation and to promote protein stabilization. Especially among the up-regulated DEGs, 10 genes were respectively identified as Hsp70 family chaperones (***FES1**, SSA2, **SSA3**, **SSA4***, and ***SSC1***) and Hsp90 family chaperones (*CDC37, **HSC82**, **HSP82**, **SSE1***, and ***STI1***). Interestingly,***SGT2*** encoded co-chaperone binds and regulates Hsp70, and ***HSP104*** encoded disaggregase interacts with Ydj1p (Hsp40) and Ssa1p (Hsp70) to rescue denatured and aggregated proteins. Cyclophilin encoded by ***CPR6*** and co-chaperone encoded by *SBA1* can bind and regulate Hsp90, while *YDJ1* is involved in regulating both the activities of Hsp70 and Hsp90. Simultaneously, large amounts of DEGs (40 genes) in the ubiquitin-dependent protein catabolic process were enhanced for degradation of damaged proteins and organelles. Thereinto, proteins encoded by 18 genes constituted subunits of the 20S and 26S proteasomes. The expression of 21 DEGs in vesicle-mediated transport was also significantly elevated after acetic acid treatment. These data suggested that acetic acid activated the ubiquitination process and the intracellular vacuolation in *S. cerevisiae*, in accordance with the above phenotypic analyses.

Up to 55 genes of ribosome, including ribosomal 40S and 60S subunits, were extensively repressed in the acetic acid-treated cells. There were 8 up-regulated DEGs (***FAL1**, **FCF1**, MTR2, REH1, REX4, RIX7, **RNH70***, and *SNM1*) involved in ribosome biogenesis, but the down-regulated *UTP22* was required for the nuclear export of tRNAs. Moreover, gene expressions of 4 DEGs (***DIA4**, EFT2, FMT1*, and *PET122*) in the translation process were down-regulated. These findings suggested that protein synthesis was also markedly repressed under the stress of acetic acid.

There are 18 DEGs involved in five mitogen-activated protein kinase (MAPK) signaling pathways in yeast^30^: high osmolarity/glycerol pathway (*CDC37, **PTP2**, SSK22* ↑, and ***PTC2**, **SMP1*** ↓), filamentous growth pathway (***FLO11*** ↑, and ***KSS1**, **YPS1*** ↓), cell wall integrity pathway (***PTP2**, **ZEO1*** ↑, and *FKS1, **GSC2**, PKH1, **YPS1*** ↓), spore wall assembly pathway (***AMA1**, **GSC2**, **SPS1*** ↓), and pheromone response pathway (*FUS1* ↑, and *FAR1, **KSS1**, **PPQ1**, STE3* ↓). Compared with other pathways, acetic acid stress had no systemic effects on the high osmolarity/glycerol pathway at the transcriptional level. There were 8 up-regulated and 8 down-regulated DEGs identified in the filamentous growth, which was a process in response to nutrient limitation, demonstrating that the filamentous growth pathway in *S. cerevisiae* was dysfunctional during acetic acid treatment. The same disorder appeared in pheromone response (5 up-regulated and 5 down-regulated genes). By contrast, the cell wall integrity (CWI) pathway was greatly changed under this condition. Although 7 up-regulated genes of cell wall mannoprotein were identified, acetic acid systematically suppressed the CWI pathway at the mRNA level. There were 24 of 28 DEGs in cell wall organization repressed after acetic acid treatment. Especially, three down-regulated genes (*FKS1, **GSC2***, and ***KRE6***) were required for the glucan biosynthesis in the cell wall. Additionally, the up-regulated ***PTP2*** was involved in the inactivation of MAPK activity in cell wall organization^31^. These results might partly explain the changes of surface morphology in the cell wall. Likewise, three key genes (***AMA1**, **GSC2***, and ***SPS1***) in the spore wall assembly (SWA) pathway were significantly down-regulated. Ascospore formation was also repressed in response to acetic acid stress, and 18 down-regulated DEGs were identified in this process. Among the DEGs, 11 genes (*ADY3, **AMA1**, **GAS2**, **GSC2**, MUM3, **QDR3**, RRT12, SMA2, **SPO73**, **SPO75***, and ***SPS1***) were responsible for the ascospore wall assembly.

Mating pheromones activate the pheromone response pathway, then induces cell cycle arrest^32^. Upon acetic acid stress, the cell cycle (29 up-regulated and 36 down-regulated genes) was in disorder in accord with the dysfunctional pheromone response pathway. Down-regulation of *FAR1* might contribute to cell cycle progression, and the changes of other two essential genes (*RAD53* and *SFH1*) likely caused the cell cycle arrest, since *RAD53* and *SFH1* were required for cell cycle arrest and progression, respectively. In that case, a large number of genes in the mitotic and meiotic cell cycle were both affected by acetic acid stress.

DNA damage was characterized in *S. cerevisiae* cells treated with acetic acid^33^, thus DNA repair was elicited in response to this stress. There were 16 DEGs in DNA repair up-regulated in the yeast cells, providing assistance to alleviate the cell death process. As shown in Table S3, there were 22 DEGs (14 up-regulated and 8 down-regulated genes) identified in PCD, including apoptosis (11 genes), autophagy (9 genes) and mitochondrial degradation (6 genes). Among them, several genes (*ATG8, ATG33, **ATG32***, and ***CDC13***) were related to multiple cell death pathways. Five up-regulated DEGs (***SNX4**, **MMM1**, ATG8, **MDM10***, and *ATG33*) were involved in mitochondrial degradation, in accordance with the phenotypic characteristics visualized by monitoring mitochondrial matrix-targeted GFP.

### Transcriptional regulation involves interaction networks among DEGs

**Figure 6:**
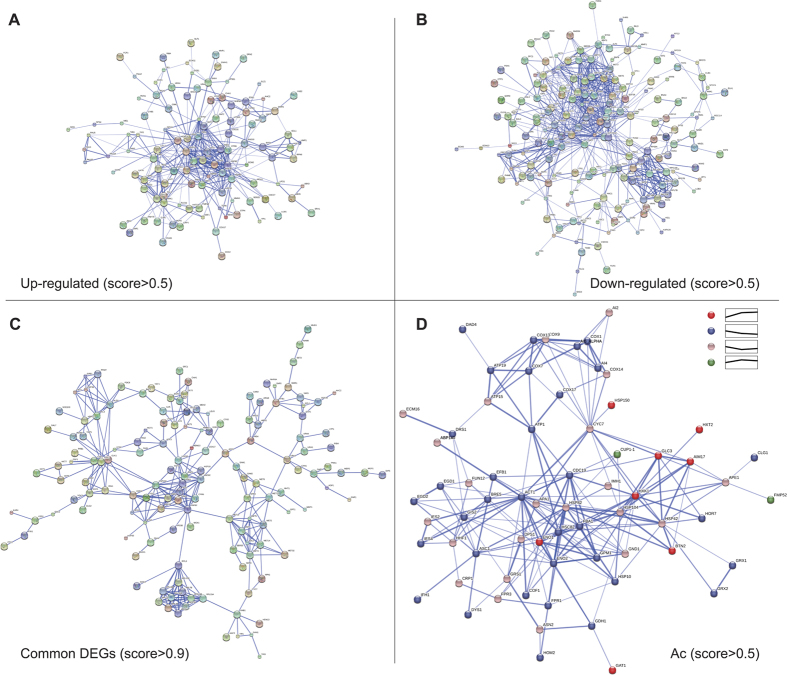
Interaction network of common DEGs at three time points. (**A**,**B**) Interaction networks of up-regulated and down-regulated DEGs with the confidence score >0.5. (**C**) Highest confidence networks of common DEGs (score >0.9). (**D**) Interaction networks of DEGs in the Ac group with four trends over time (score >0.5). Interactions are indicated by edges, with thicker edges having stronger associations.

The interactions of the identified DEGs are integrated and predicted in the STRING database (http://stringdb.org/)^34^. Figure 6A–C show the different interaction networks of up-regulated (113), down-regulated (183), and common (145) DEGs at three time points, respectively. In up-regulated DEGs, most heat shock protein genes (such as *HSP82, HSC82, HSP104, HSP42, HSP78, SSA4, STI1, HSP60, SSE1, SSA3*, etc.) have close and extensive interactions (confidence score >0.5). By contrast, the interactions (confidence score >0.5) between the down-regulated genes are mainly clustered into two groups, one for a number of closely interrelated genes (such as *GLT1, ARO1, MET5, HIS4, SAM1, MET10, MET14, STR3, GAP1, ILV5, MET32, ALD3, MET2, SAM2, MET17, MET3, MET6*, etc.) related to amino acid metabolism, and another one for interrelated ribosome genes (including *RPL3, RPS9A, RPL4B, RPL18B, RPL15A, RPL1B, RPS14B, RPL22B, RPL31B, RPL7B, RPS7B*). As mentioned above, gene expressions involved in heat shock protein, amino acid metabolism, and ribosome are affected differently by acetic acid.

To identify the potential regulatory genes induced by acetic acid, the interaction network of common DEGs over time is plotted in Fig. 6C, with the highest confidence score of more than 0.9. Clearly, there are four dominant biological processes of highly interrelated genes: heat shock protein, amino acid metabolism, ribosome, and carbohydrate metabolism (*HXK1, GLK1, ALD3, GSY1, TKL2, PRM15 (PGM3*), *EMI2, HXT6, HXT7, HXT1, DSF1*, etc.). *HSP82* is predicted to be specifically associated with the ribosome genes, thus the interaction between *HSP82* and *RPL3* may play a vital role in the cross-talk between heat shock protein and ribosome. Similarly, *HSP104* and *HSP42* are both closely related to the genes in carbohydrate metabolism. The close interaction also exists between heat shock protein and amino acid metabolism; therefore, *HSP82, HSC82,* and *HSP60* may act as the potential regulatory genes.

### Transcriptional responses show temporal- and spatial-specific expression in acetic acid treated cells

The core functions and pathways in response to acetic acid have been identified, but the temporal and spatial changes of gene expression over time under this stress remains unclear. Enrichment of MIPS functional categories from all of the DEGs at different times contributes to the assessment of the transcriptional changes upon acetic acid stress. The up-regulated and down-regulated genes are functionally categorized in Table S4, respectively. At different times, the ‘Protein with binding function or cofactor requirement (structural or catalytic)’ and ‘Cell rescue, defense and virulence’ categories are overrepresented for the common genes up-regulated in response to acetic acid, and ‘Metabolism’ is overrepresented for the down-regulated genes. These findings demonstrate that most transcriptional events are involved in these three categories under acetic acid stress, and take place throughout the entire time. On the one hand, the yeast cells were trying to rescue themselves and put up a defense against this stress in various ways. On the other hand, intracellular metabolism was continuously suppressed, especially in amino acid and carbohydrate metabolism.

Interestingly, the other overrepresented categories are obviously temporal- and spatial-specific. At 45 min, a large number of genes in ‘Transcription’ and ‘Protein synthesis’ were up-regulated, meanwhile ‘Cellular transport, transport facilities and transport routes’, ‘Cell type differentiation’, ‘Interaction with the environment’, and ‘Energy’ were down-regulated. Subsequently, ‘Protein fate (folding, modification, destination)’ and ‘Cell cycle and DNA processing’ were both enhanced, but gene transcription in ‘Cellular transport, transport facilities and transport routes’ and ‘Protein synthesis’ were reduced at 120 min. Then, ‘Regulation of metabolism and protein function’ was improved as well as ‘Protein fate (folding, modification, destination)’ at 200 min. However, ‘Protein with binding function or cofactor requirement (structural or catalytic)’ and ‘Protein synthesis’ were greatly suppressed at the same time. Obviously, the biological functions and pathways in the yeast cells follow the spatial and temporal order under acetic acid stress.

In the Ac group, 73 DEGs at three time points were selected (FDR < 0.05), of which 36 genes were continuously down-regulated and 9 genes were up-regulated. This further confirmed that more genes were inhibited by acetic acid. KEGG classification revealed glycolysis (*ENO2, GPM1, CDC1*9, and *FBA1*) and oxidative phosphorylation (*COX1, AI5_ALPHA, AI4, ATP1, ATP19, COX7, COX13*, and *COX17*) were the main biological processes with a downward tendency, suggesting that both processes were mostly inhibited over time. The interactions of 66 selected genes were plotted using the STRING database (Fig. 6D). *ACT1* gene encoding actin was interrelated with 25 genes (the confidence score >0.5), thus it might be an essential node in programmed cell death induced by acetic acid. Additionally, *HSP82* (18 interactions) and *HSC82* (14 interactions) both encode the chaperones of the Hsp90 family, and function respectively to promote cell survival and pro-death in acetic acid-induced apoptosis^35^. As shown in Fig. 6D, the difference in transcriptional changes implied their distinct roles with the time effect in the process.

### NMetabolic fluxes in yeast cells are altered during acetic acid treatment

As mentioned above, the central metabolic pathway was greatly changed at the mRNA level during acetic acid treatment. The intracellular metabolites were further analyzed using gas chromatography-mass spectrometry (GC-MS). Total ion current (TIC) chromatograms of the CK and Ac groups were presented in Fig. S5, and 89 metabolites in *S. cerevisiae* were compared at different times. Differential intracellular metabolites showing a significant difference (with the threshold |log2(fold change)| > 1, *P* value < 0.05) were summarized in Table S5. It was demonstrated that acetic acid had varying impacts on amino acid metabolism, carbohydrate metabolism, and lipid metabolism. Hereinafter, the common differential metabolites at different times are marked in bold.

Compared with untreated cells, all detected amino acids (**alanine**, **asparagine**, **aspartic acid**, glutamine, **glycine**, **isoleucine**, **leucine**, lysine, **methionine**, **ornithine**, **phenylalanine**, **proline**, **serine**, **threonine**, **tyrosine**, and **valine**) were dramatically reduced in the yeast cells treated with acetic acid at more than one time point (fold change >2, *P* < 0.05, two-tailed *t* test). The related metabolites (2-aminobutyric acid, **4-aminobutyric acid**, **pyroglutamic acid**, and **uracil**) were consistent with the decrease of amino acids. These results further supported that the uptake and biosynthesis of amino acids were suppressed upon acetic acid stress.

By contrast, glycometabolism was significantly altered by acetic acid addition. The accumulations of four metabolites (mannitol, inositol-3-phosphate, frucose-6-phosphate, and glucose-6-phosphate) were varied at different times. However, there were three important monosaccharides (galactose, fructose, and glucose) that increased at more than one time point. Although **citric acid** and **glyceric acid** were higher in the Ac group than in the control, the accumulation of **succinic acid** and other related metabolites was definitely suppressed under acetic acid stress. Obviously, acetic acid blocked biosynthesis of amino acids from glycolysis, TCA cycle and other pathways. In addition, the contents of five long-chain fatty acids (linoleic acid, oleic acid, palmitelaidic acid, dodecanoic acid, and 11-*cis*-octadecenoic acid) in the yeast cells were mainly increased in response to acetic acid stress.

KEGG functional classification of differential metabolites in the Ac group (Table S5) indicated that biosynthesis of amino acids was still negatively influenced by acetic acid over time. There were a large amount of down-regulated metabolites in the metabolic pathways. Secondly, many metabolites related to carbohydrate metabolism were reduced in acetic acid-treated cells with the progression of time. These data verified the above findings derived from RNA-Seq analysis.

### Acetylation imbalance aggravates cell death under acetic acid stress

Histone acetylation is a protein post-translational modification conserved in yeast, and mediates acetyl-Co A metabolism and cellular signaling^36^. Acetylation of metabolic enzymes regulates cell growth and metabolic flux^37^. RNA-Seq data showed acetic acid enhanced histone acetylation in the yeast cells, but its impact on cell death is largely unknown. There were 10 DEGs involved in histone acetylation and deacetylation (*ADA2, AHC2, ESA1, EPL1, HPA2, HOS1, HST3, SGF29, YAF9*, and *SET2*) that were further investigated. First, we overexpressed all of the genes in yeast cells to detect the cell mortality rate of the transformed strains compared with the control. It was found that overexpression of most genes (*ADA2, AHC2, ESA1, EPL1, YAF9*, and *SET2*) increased cell death induced by acetic acid (150 mM) in SC2 medium [0.67% (w/v) YNB, 2% (w/v) D-glucose, 0.008% (w/v) histidine, 0.02% (w/v) leucine, 0.003% (w/v) lysine and 0.032% (w/v) uracil, pH 3.0], whereas only the overexpression of *HPA2* led to the opposite phenotype (Fig. S6A). Thereinto, *SET2* encodes histone methyltransferase and signals for histone deacetylation^38^.

To further determine the relationship between cell death and acetylation balance, we compared the cell death between wild-type (WT) and mutants of genes in histone acetylation and deacetylation. Interestingly, the deletion of six genes (*ADA2, AHC2, HPA2, HOS1, SGF29*, and *YAF9*) also enhanced cell death upon acetic acid stress (Fig. S6B). Apart from these six genes, we tried to delete the *ESA1* or *EPL1* gene in yeast, but we were not successful in obtaining the haploid mutants. Given the lethality of the *ESA1* and *EPL1* knockout^39^,^40^, we used the diploid mutants *esa1Δ*/*ESA1, epl1Δ*/*EPL1* and wild-type strain from EUROSCARF for further research. The first two mutants were constructed by knocking out a single allele of the target genes in the diploid WT. As shown in Fig. S6C, no significant differences were observed in the death rate between the diploid mutants and wild-type (*P* > 0.05, two-tailed *t* test). It was probably because of the intact allele making up for the gene expression of the deleted one. In balance, unidirectional changes of histone acetylation and deacetylation are likely to be crucial to cell death during acetic acid treatment. To check the above inference, sodium butyrate (SB), a typical histone deacetylase inhibitor in yeast^41^, was used to change the acetylation balance before acetic acid treatment. As presented in Fig. S6D, SB increased the cell death rate in a dose-dependent manner. Pre-incubation with 10 mM SB had no significant effect on cell death in acetic acid-treated cells (*P* > 0.05, two-tailed *t* test); however, the cell death induced by acetic acid was greatly enhanced when the concentration of SB was above 20 mM. Overall, acetylation imbalance, especially over-enhanced histone acetylation, would aggravate cell death upon acetic acid stress.

## Discussion

Acetic acid negatively influences yeast growth and ethanol yields^4^, and even triggers programmed cell death in *S. cerevisiae*^10^. A comprehensive understanding of stress responses and cell death in *S. cerevisiae* under acetic acid stress is crucial for developing robust yeast strains in ethanologenic fermentation. A number of DEGs (≤200) were discovered upon exposure to acetic acid under different conditions based on the transcriptome datasets^15^,^16^,^17^,^18^,^19^,^20^, five of which were conducted by DNA microarray analysis. Unlike the studies of Lee *et al*.^15^, we chose more severe conditions with higher concentrations of acetic acid (150 mM, about 0.9% acetic acid) and lower culture pH (3.0) than that of Lee *et al*. (0.6% acetic acid, pH 4.5). Thus, we revealed more transcriptional changes of DEGs (295 up-regulated and 427 down-regulated cross-DEGs) under the extreme conditions. The transcriptional responses in yeast cells followed the spatial and temporal order in response to acetic acid stress. There are some similar findings in MIPS functional categories between our data at 45 min and the one of Lee *et al*.^15^. For instance, ‘Protein synthesis’ and ‘Protein with binding function’ were up-regulated, while ‘Energy’ and ‘Transport’ were down-regulated. It might be because acetic acid treatment (150 mM, pH 3.0) in the early stage caused some similar transcriptional responses with a relatively moderate stress. Nonetheless, there were still opposite results in ‘Metabolism’ and ‘Cell rescue, defense, virulence’, and more differences in other aspects.

Although it has been reported that the medium with lower pH aggravates PCD in yeast^5^, there is still no clear understanding of this phenomenon. In this study, we found that yeast cells are able to tolerate acidic conditions with a low environmental pH, ranging from 6.0 to 3.0. The cells untreated with acetic acid maintained a neutral pH (about 7.0) in cytosol and a weak alkaline pH (about 7.8) in the mitochondrial matrix at a low culture pH (3.0). In contrast, acetic acid (≥30 mM) induced cell death and mitochondrial degradation in a dose-dependent manner when the culture pH remained at 3.0. This suggested an intrinsic mechanism underlying acetic acid induced cell death, differing from the effect of extracellular pH. The undissociated acetic acid gets inside cells by facilitated and passive diffusion^42^, and then dissociates to generate protons and anions in the intracellular environment at a neutral pH. *In situ* pHluorin measurements revealed that cytosolic and mitochondrial pH dropped to below 4.0 and 5.3 at 120 min, respectively, showing serious intracellular acidification. A series of transcriptional events were initially induced after acetic acid treatment. Correspondingly, the expression of key genes involved in proton export (*PMA1, PMA2*, etc.) was significantly down-regulated. Although intracellular pH can be restored at a low concentration of acetic acid^6^, the restorability was obviously blocked by high concentrations of acetic acid. Especially, the down-regulated *VMA1* gene encoded the V-type ATPase in various organelle membranes, which was required in proton transfer from cytosol to organelles, including vacuole, endosomes, and late Golgi apparatus^27^. The up-regulated *HSP30* gene had a negative effect on the plasma membrane H^+^-ATPase Pma1p^43^, which exported protons out of the cell. Together, inhibition of proton export intensified the acidification in cytosol and mitochondrial matrix. Since the binding and conformational stability of proteins are dependent on intracellular pH^27^, this serious acidification could denature large amounts of proteins, and aggravated the damage to the plasma membrane and organelles over time, then activated a cascade of cell death.

**Figure 7:**
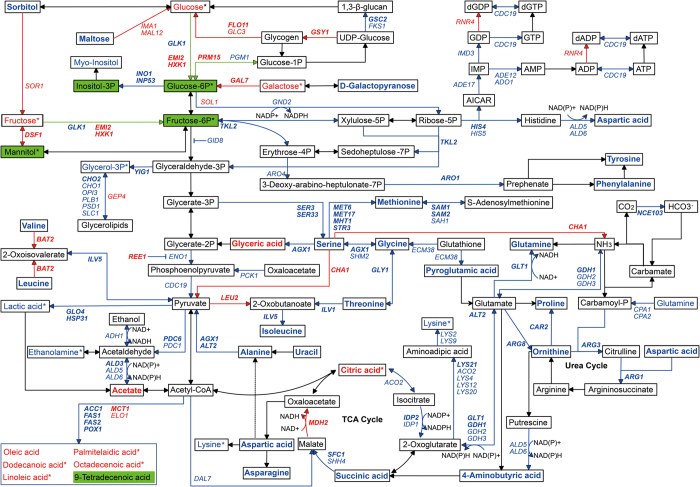
The central metabolic pathways of yeast cells in response to acetic acid. Up-regulated genes and metabolites at more than two time points are highlighted in red, while down-regulated genes and metabolites are highlighted in blue. Common DEGs and metabolites at three time points are emphasized in bold. The metabolites showing both significant increase and decrease are highlighted in green. *P value ≥ 0.05, two-tailed *t* test.

The redox homeostasis between NAD(P)H and NAD(P) plays a major role in the modification of the metabolic flux in yeast^44^. Intracellular pH is considered to have a crucial influence on the oxidation-reduction potential of specific reductases and dehydrogenases^27^. Acetic acid-induced acidification greatly suppressed the expression of many genes involved in redox transformation from NAD(P) to NAD(P)H. Consequently, the redox homeostasis was disrupted by this acid, thus the metabolic process was dramatically inhibited. Additionally, it was found that acetic acid severely reduced adenosine triphosphate (ATP) levels and the gene expression of some nutrient transporters^42^, resulting in severe amino acid starvation^14^. Our transcriptomic analysis further revealed this acid not only decreased gene expression of almost all identified permeases and transporters located in intracellular membranes, but it also reduced biosynthesis of amino acids at the mRNA level. A change of intracellular metabolites is a direct mirror of transcriptional regulation and protein function in cells^45^. The findings at the transcriptional level were emphasized by the metabolomics analysis (Fig. 7). Simultaneously, several genes in amino acid catabolism were significantly up-regulated. All of these proved that the uptake and biosynthesis of amino acids were comprehensively blocked by acetic acid at the both transcriptional and metabolic levels.

In accordance with the six up-regulated genes of hexose transporters, gene expression upstream of carbohydrate metabolism was enhanced in response to acetic acid. The main upstream metabolites also accumulated in the treated cells. In contrast, gene expression and metabolites in downstream of carbohydrate metabolism were decreased (Fig. 7). Under certain conditions, acetate might be consumed in the TCA cycle and fatty acid metabolism after conversion into acetyl Co-A^7^,^9^. Citric acid also accumulated in the acetic acid-treated cells under the given conditions, but the subsequent metabolic process in TCA cycle was suppressed. Furthermore, contrary to the substantial accumulation of long-chain fatty acids, most genes in lipid metabolism and the key genes (*FAS1, FAS1*, and *ACC1*) in fatty acid biosynthesis were transcriptionally down-regulated. The up-regulated genes (*MCT1* and *ELO1*) in fatty acid elongation and degradation of lipid droplets might explain this difference. The dissociated acetate could not be metabolized via TCA cycle and fatty acid synthesis, thus intracellular acetylation was largely intensified. Intracellular acidification caused by dissociated protons has been reported^5^,^6^, but the fate and impact of dissociated acetate remain unclear. As demonstrated in our work, cell death under acetic acid stress was respectively enhanced by the overexpression or deletion of six different DEGs involved in histone acetylation and deacetylation. The increased cell death was also observed when the acetylation balance was changed by the histone deacetylase inhibitor (sodium butyrate). Therefore, the acetylation imbalance likely aggravated acetic acid-induced cell death. These experimental works might be not perfect, but provided important evidences for further research.

Another result of intracellular acidification is that a large number of proteins and organelles are impaired, protein misfolding and denaturation are increased by acetic acid. On the one hand, this would promote gene expression of the heat shock protein family and protein folding in endoplasmic reticulum. In view of the anti-apoptotic activities^35^,^46^, involvement of the heat shock proteins (like Hsp70 and Hsp90) possibly contributes to the alleviation of acetic acid stress. Consistently, many heat shock proteins are coordinated to refold and reactivate denatured and aggregated proteins upon acetic acid stress^47^. On the other hand, acetic acid greatly activated the ubiquitin-dependent protein catabolic process and the vesicle-mediated transport. These were in agreement with the vacuolation of cytoplasm observed by TEM, and organelles rapidly disintegrated or collapsed under this stress. These also resulted in mitochondrial degradation and loss of viability in the yeast cells. Protein synthesis was also significantly reduced in response to acetic acid stress, which was accompanied by the decreased gene expression of numerous ribosomal 40 S and 60 S subunits.

MAPK pathways, especially the CWI pathway, have been reported to affect yeast tolerance to various environmental stresses^48^,^49^; however, the transcriptional responses to acetic acid stress remain largely unknown. As the first barrier to resist external stress in yeast, the cell wall protects cells from various injuries. Modulation of the CWI pathway was regard as an effective strategy to increase acetic acid resistance^50^. Maintenance of glucan synthase activity and cell integrity were required for yeast tolerance to this weak acid at pH 4.5^51^. During acetic acid treatment, TEM and SEM images displayed that the treated cells became fragile with a thinner cell wall. Consistent with this phenotype, a large number of DEGs in the CWI pathway and cell wall organization were down-regulated by a high concentration of acetic acid at a low pH. Among the down-regulated DEGs, 9 genes, namely *CWH41, FKS1, GAS2, GSC2, KRE6, PSA1, SCW11, SIM1*, and *YUR1*, were involved in carbohydrate metabolism, which was essential for cell wall remodeling. It implied a potential link between the CWI pathway and carbohydrate metabolism in response to acetic acid stress. Moreover, the SWA pathway and ascospore wall assembly were suppressed at the transcriptional level under this condition, thus inhibiting ascospore formation in yeast. In addition, mitosis and meiosis were also disturbed by acetic acid, as was the dysfunctional pheromone response pathway.

Overall, it was not a simple physiological process in cell death induced by acetic acid, and yeast cells were likely to have multiple responses upon acetic acid stress. Various biological pathways could be connected by protein–protein interactions. The interaction networks predicted using STRING v10 have revealed the potential hubs of intracellular processes upon acetic acid treatment (Fig. 6). The heat shock protein family is predominant in up-regulated DEGs, while amino acid metabolism and ribosome are respectively interrelated in down-regulated DEGs. Several heat shock protein genes (*HSP82, HSC82, HSP42, HSP60*, and *HSP104*) are suggested as potential regulatory factors. Intriguingly, *HSP82* and *HSC82* have been proven to have distinct roles in the cellular modulation of cell death induced by acetic acid^35^. Nevertheless, these potential regulatory genes still need to be further confirmed.

In acetic acid-treated cells, glycolysis and oxidative phosphorylation were found to be suppressed over time. Therefore, metabolic transformation and energy supply might be blocked by acetic acid as time progresses. Correspondingly, the metabolites associated with the biosynthesis of amino acids and carbohydrate metabolism were decreased in the treated cells over time. The interaction network indicated that the *ACT1* gene was potentially a crucial node in this process. In particular, actin cytoskeleton encoded by *ACT1* was identified to be a major target in apoptotic processes, and it was closely tethered to the apoptotic response in *S. cerevisiae*^52^. In addition, *HSP82* and *HSC82* as well were interrelated with a large number of other genes, thus they may be key genes with time effect during acetic acid-induced cell death.

The fundamental issue of PCD in unicellular organisms is a vexing one. Some evidence explains why PCD occurs in unicells^10^,^53^, but no comprehensive investigation has been done on mechanisms and the genetic regulation of PCD in a model organism like *S cerevisiae* based on the physiologic, genetic, transcriptomic, and metabolomic data. This work helps address our general understanding of genetic mechanisms for PCD in another model organism like *Chlamydomonas*^54^.

## Conclusions

Under low pH and nitrogen conditions, acetic acid has systemic effects on physiological, transcriptomic, and metabolomic responses in *S. cerevisiae*. It differs from the impact of change in extracellular pH. A high concentration of acetic acid was dissociated in neutral cytosol after entering the cells. On the one hand, the dissociation caused intracellular acidification in both the cytoplasmic and mitochondrial matrix. Gene expression in maintaining intracellular pH and redox homeostasis was significantly suppressed. This stress greatly changed the global metabolism. Nutrient uptake was inhibited with the down-regulation of a series of permeases and transporters. Biosynthesis of amino acids and related carbohydrate metabolism were comprehensively decreased at both transcriptional and metabolic levels. Long-chain fatty acids were accumulated in this process, but the key genes in fatty acid biosynthesis and most DEGs in lipid metabolism were down-regulated. On the other hand, the dissociated acetate largely intensified the intracellular acetylation level in yeast, and the acetylation imbalance aggravated cell death induced by acetic acid. Additionally, large amounts of proteins and cellular structures were denatured and damaged under this stress, which activated protein folding and stabilization, ubiquitin-dependent protein catabolic process, and vesicle-mediated transport. In contrast, protein synthesis was repressed by acetic acid owing to the reduced expression of numerous ribosomal subunits. The CWI pathway and cell wall organization were highly suppressed in response to acetic acid stress, and the cell cycle was also disturbed under this condition. Several heat shock protein genes were predicted as potential regulatory genes, which need further investigation. The findings in this study also demonstrated that the global responses in yeast have temporal- and spatial-specific effects under acetic acid stress.

## Methods

### Strains, culture conditions and acetic acid treatment

Yeast strains used in this work are listed in Table S6. To construct the plasmids for overexpression, the vector pESC-ura (Agilent Technologies) was inserted with different target genes using genomic DNA of W303-1B as template. All pESC plasmids containing a target gene were constructed by homologous recombination using ClonExpress™ II One-step Cloning Kit (Vazyme Biotech, Nanjing, China). W303-1B was transformed with pYES-ACT-pHluorin or pYES-ACT-mtpHluorin (gifts from Dr. Gertien J. Smits) for measuring pH_i_^24^, and was transformed with pYX232-mtGFP (a gift from Benedikt Westermann, Addgene plasmid # 45052) to detect mitochondrial degradation^25^. Yeast cells were transformed by the lithium acetate method. Correctness of the transformed strains was verified using PCR analysis and LSM 780 confocal microscope (Carl Zeiss MicroImaging, Göttingen, Germany). The primers and restriction enzymes for plasmid construction are shown in Table S7.

The yeasts were grown in YPD medium (1% (w/v) yeast extract, 2% (w/v) peptone, 2% (w/v) D-glucose) for non-selective propagation. W303-1B was grown in SC1 medium^55^, and BY4742 and BY4743 were grown in SC2 medium for selective propagation^26^. The transformed strains were grown in selective media lacking appropriate amino acids or nucleotides. For strains harboring a pESC plasmid, 2% galactose (SG) was used for induction expression instead of D-glucose. All deletion strains were maintained in YPD medium containing 200 μg/ml G418. Unless otherwise mentioned, freshly grown cells of exponential phase were harvested for cultivation or treatment after incubation with an agitation of 180 rpm at 28 °C in the selective medium.

For acetic acid treatment, yeast strains in the exponential phase were harvested and suspended in SC or SG broth at pH 3.0 (set with HCl) containing 150 mM of acetic acid, and cultured for different times at 28 °C, in an orbital shaker at 160 rpm, with a ratio of flask volume/medium of 5:1. After washing the cells with sterile distilled water, a small fraction was diluted to a suitable concentration and counted with a Scepter™ 2.0 Cell Counter (Merck, Darmstadt, Germany). The initial inoculum concentration was 1 × 10^7^ cells/ml. At least three independent experiments were carried out for each condition unless otherwise stated. For RNA sequencing and metabolomic analysis, the samples were taken at 45 min, 120 min, and 200 min after acetic acid treatment. The untreated cells served as the controls.

### Cell survival and mitochondrial degradation

Cell growth of treated and untreated cells was followed by measuring culture OD660. Cell viability with treatment for 3 h was assayed by counting colony-forming units (CFU) on YPD agar plates after 2 d at 30 °C. Mitochondrial degradation was measured by quantifying mitochondrial GFP fluorescence using a FC500MCL flow cytometer (Beckman Coulter, USA)^11^.

### PCD assay, TEM and SEM observation

The Annexin V-FITC/PI apoptosis kit (Lianke, Hangzhou, China) was used to evaluate acetic acid-induced cell death by flow cytometry. Briefly, yeast cells were first collected and digested with 20 U/ml lyticase (Sigma-Aldrich) in sorbitol buffer (1.2 M sorbitol, 0.5 mM MgCl_2_, 35 mM K_2_HPO_4_, pH 6.8) at 30 °C for 50 min. 1 × 10^6^ cells were washed and resuspended in 500 μl 1 × binding buffer containing 1.2 M sorbitol, then co-stained with Annexin V and PI at room temperature for 10 minutes in the dark before determination.

To investigate the impact of acetylation imbalance on cell death induced by acetic acid, sodium butyrate (SB), a well-known histone deacetylase inhibitor^41^,^56^, was used to alter the acetylation balance by suppressing histone deacetylation. Cell death was quantified by flow cytometry using propidium iodide (PI) staining. Cells were incubated with 0, 10, 20, and 40 mM SB in an orbital shaker for 30 min at 28 °C and 160 rpm before treatment with 150 mM acetic acid for 120 min. The collected cells were resuspended in 500 μl PBS with 5 μg/ml PI for 10 min at 28 °C in the dark, and then they were analyzed by flow cytometry.

For TEM and SEM, yeast cells were harvested at different times after acetic acid treatment. The intracellular morphology was analyzed using a JEM-1230 transmission electron microscope (JEOL, Japan). Extracellular morphological analysis was performed using a S-3000N electron microscope (Hitachi, Japan).

### Measurements of pH_cyt_ and pH_mit_

The measurements of pH_cyt_ and pH_mit_ were respectively taken using the pH-sensitive ratiometric pHluorin in cytosol and mitochondria as references^24^,^28^. The strains expressing pHluorin during exponential growth were first transferred to Costar black 96-well microtitrer plates (Corning, USA), and incubated in 200 μl Verduyn medium^57^ containing 5 g/L (w/v) glucose, with or without treatment of 150 mM acetic acid (pH 3.0) for different times. The ratios of pHluorin emission at 512 nm by 390 and 470 nm excitation (R_390/470_) were measured using a SpectraMax M5 Multi-Mode Microplate Reader (Molecular Devices, USA). The calibration curves of R_390/470_ against the pH were plotted after background subtraction of untransformed cultures^24^. Before that, yeast cells were permeabilized with 100 μg/mL digitonin in PBS for 10 min, then washed and resuspended in phosphate-citrate buffers ranging from pH 3.5 to 8.0. pH_cyt_ and pH_mit_ can be accurately calculated according to the calibration curves (r^2^ = 0.996 and 0.993). To analyze pHluorin expression in cytosol and mitochondria, the green fluorescence was visualized by confocal microscopy.

### RNA sequencing (RNA-Seq) analysis

Yeast cells were harvested as rapidly as possible for total RNA extraction at the three time points listed above. Aliquots of 4 × 10^7^ cells were taken at each time point before centrifugation at 1000 g for 5 min at 4 °C. Total RNA was immediately extracted with the RNeasyMini Kit (Qiagen, Hilden, Germany) and treated with the RNase-Free DNase Set (Qiagen) to remove any contaminating genomic DNA, according to the manufacturer’s protocols. RNA quality and concentration were assessed using the Agilent 2200 TapeStation system (Agilent Technologies, Santa Clara, USA) and NanoDrop 2000 Spectrophotometer (Thermo Fisher Scientific, Waltham, USA), respectively. Duplicate RNA samples with the best quality were selected for transcriptional analysis from three independent experiments for each condition.

RNA-Seq was performed by RiboBio Co., Ltd. Briefly, libraries for RNA-Seq were prepared with TruSeq^®^ RNA LT/HT Sample Prep Kit (Illumina, USA) following the manufacturer’s protocol. The purified libraries were assessed using the Agilent 2200 TapeStation and Qubit^®^2.0 (Life Technologies,USA), and subsequently sequenced on an Illumina HiSeq 2500 with 2 × 100-bp paired-end reads. Raw reads were trimmed for clean data and mapped to the S288c genome with Tophat (v2.0.10)^58^. The gene expression abundance was normalized by FPKM (fragments per kilobase of exon per million fragments mapped) using Cufflinks (v2.2.1)^59^. Differentially expressed genes (DEGs) were identified, with the threshold |log2(Fold change)| > 1 and *q*-value < 0.05 as the criteria of significant gene expression difference.

Enrichment of GO terms and KEGG pathways was analyzed based on the identified DEGs. Enrichment of functional categories among DEGs was performed with the MIPS Functional Catalogue (http://mips.helmholtz-muenchen.de/funcatDB/). Specific gene functions and biological pathways were annotated according to SGD (http://www.yeastgenome.org) and UniProt (http://www.uniprot.org/). The interaction networks of DEGs were obtained using the STRING v10 database (http://string-db.org/).

### qPCR verification for transcriptomic data

To verify the transcriptomic findings, 15 genes were selected for qPCR analysis. The cDNA synthesis and elimination of genomic DNA contamination were performed by the HiScript^®^ Q RT SuperMix for qPCR (+gDNA wiper) (Vazyme Biotech). Subsequently, qPCR measurement was carried out with ChamQ SYBR qPCR Master Mix (Vazyme Biotech) on a CFX Touch™ Real-Time PCR Detection System (Bio-Rad, Hercules, USA) following the manufacturer’s description. The *ACT1* gene was used as a reference, and the fold change was quantified by the 2^−ΔΔCt^ method. Specific primers were designed and confirmed by melting curve analysis. All primers used in qPCR are listed in Table S8.

### Metabolite extraction and GC-MS analysis

Samples (approximately 100 mg of fresh weight) collected by centrifugation were added to 10 mL ddH_2_O, and mixed quickly with 25 mL of 60% (v/v) methanol quenching solution pre-cooled to −40 °C. The pellets were washed with ice-cold ddH_2_O and then stored in liquid nitrogen until extraction after centrifugation at 3000 g for 5 min at 4 °C. Herein, intracellular metabolites were extracted according to the protocol described by Sellick *et al*.^60^. Six independent replicates were used to perform metabolomic analysis for each condition.

The derivatized samples (1.0 μl) were analyzed using an Agilent 7890 A GC/5975 C MS system (Agilent Technologies), and separated with a HP-5MS capillary column (5% phenyl methyl silox: 30 m × 250 μm i.d., 0.25-μm, Agilent J&W Scientific, Folsom, CA, USA) and a helium carrier gas flow rate of 1 ml/min. The split ratio was 1:20 (v/v). Temperatures of injection, ion source and interface were 280 °C, 250 °C and 150 °C, respectively. The temperature gradient was programmed at 40 °C for 5 min, 10 °C/min to 300 °C, and held for 5 min. Full scan mass spectra were obtained by electrospray ionization (ESI) at 70 eV, ranging from 35 to 780 (m/z).

Identification of metabolites was performed by searching the NIST 2008 mass spectra library and Golm Metabolome Database (http://gmd.mpimp-golm.mpg.de/), and 21 compounds (including alanine, valine, leucine, isoleucine, proline, glycine, serine, threonine, methionine, aspartic acid, glutamine, ghenylalanine, asparagine, lysine, tyrosine, arabinose, fructose, galactose, glucose, mannitol, and cellobiose) were further confirmed by authentic standard compounds. Raw GC/MS files were converted into the CDF format, and were subsequently pretreated by XCMS software (www.bioconductor.org/).

## Additional Information

**How to cite this article:** Dong, Y. *et al*. RNA-Seq-based transcriptomic and metabolomic analysis reveal stress responses and programmed cell death induced by acetic acid in *Saccharomyces cerevisiae.* *Sci. Rep.* **7**, 42659; doi: 10.1038/srep42659 (2017).

**Publisher's note:** Springer Nature remains neutral with regard to jurisdictional claims in published maps and institutional affiliations.

## Supplementary Material

Supplementary Tables and Figures

Supplementary Table S5
