# Supplementary material for: RNA-Seq-based transcriptomic and metabolomic analysis reveal stress responses and programmed cell death induced by acetic acid in Saccharomyces cerevisiae
Source: Sci Rep. 2017 Feb 17;7:42659. doi: 10.1038/srep42659 (PMC5314350; doi:10.1038/srep42659)

RNA-Seq-based transcriptomic and metabolomic analysis reveal stress responses and programmed cell death induced by acetic acid in *Saccharomyces cerevisiae*

Yachen Dong, Jingjin Hu, Linlin Fan, Qihe Chen*

Department of Food Science and Nutrition, Key Laboratory for Food Microbial Technology of Zhejiang Province, Zhejiang University, 866 Yuhangtang Road, Hangzhou 310058, China

* Correspondence and requests for materials should be addressed to Q.H.C. (chenqh@zju.edu.cn).

**Supplementary Tables**

**Table S1.** Summary of RNA-seq data.

| Samples | Data | Total reads | Clean reads | Clean_Q20^a^ | Clean_Q30^b^ | Mapped reads^c^ |
| --- | --- | --- | --- | --- | --- | --- |
| CK45-1 | sample2-read1 | 18,987,450 (100%) | 18,103,006 (95.34%) | 99.35% | 97.19% | 17,344,351 (47.90%) |
|  | sample2-read2 | 18,987,450 (100%) | 18,103,006 (95.34%) | 99.27% | 96.64% | 17,358,325 (47.94%) |
| CK45-2 | Sample3-read1 | 20,654,219 (100%) | 19,441,584 (94.13%) | 99.36% | 97.25% | 18,591,155 (47.81%) |
|  | Sample3-read2 | 20,654,219 (100%) | 19,441,584 (94.13%) | 99.25% | 96.56% | 18,608,271 (47.86%) |
| Ac45-1 | Sample4-read1 | 18,607,099 (100%) | 17,711,402 (95.19%) | 99.34% | 97.14% | 16,975,360 (47.92%) |
|  | Sample4-read2 | 18,607,099 (100%) | 17,711,402 (95.19%) | 99.26% | 96.59% | 16,989,348 (47.96%) |
| Ac45-2 | Sample6-read1 | 17,838,978 (100%) | 16,950,909 (95.02%) | 99.35% | 97.18% | 16,296,781 (48.07%) |
|  | Sample6-read2 | 17,838,978 (100%) | 16,950,909 (95.02%) | 99.24% | 96.54% | 16,300,458 (48.08%) |
| CK120-1 | Sample7-read1 | 16,739,498 (100%) | 15,814,057 (94.47%) | 99.37% | 97.29% | 15,125,460 (47.82%) |
|  | Sample7-read2 | 16,739,498 (100%) | 15,814,057 (94.47%) | 99.25% | 96.57% | 15,127,171 (47.83%) |
| CK120-2 | Sample8-read1 | 16,704,888 (100%) | 15,845,645 (94.86%) | 99.32% | 97.01% | 15,046,119 (47.48%) |
|  | Sample8-read2 | 16,704,888 (100%) | 15,845,645 (94.86%) | 99.21% | 96.37% | 15,047,118 (47.48%) |
| Ac120-1 | Sample10-read1 | 16,452,251 (100%) | 15,559,777 (94.58%) | 99.38% | 97.34% | 14,920,859 (47.95%) |
|  | Sample10-read2 | 16,452,251 (100%) | 15,559,777 (94.58%) | 99.23% | 96.53% | 14,915,316 (47.93%) |
| Ac120-2 | Sample11-read1 | 17,737,146 (100%) | 16,800,379 (94.72%) | 99.37% | 97.28% | 16,118,317 (47.97%) |
|  | Sample11-read2 | 17,737,146 (100%) | 16,800,379 (94.72%) | 99.23% | 96.50% | 16,110,010 (47.95%) |
| CK200-1 | Sample13-read1 | 25,091,995 (100%) | 23,922,474 (95.34%) | 99.26% | 95.74% | 23,010,445 (48.09%) |
|  | Sample13-read2 | 25,091,995 (100%) | 23,922,474 (95.34%) | 99.30% | 95.82% | 23,037,329 (48.15%) |
| CK200-2 | Sample14-read1 | 18,725,385 (100%) | 17,745,695 (94.77%) | 99.32% | 96.84% | 16,942,665 (47.74%) |
|  | Sample14-read2 | 18,725,385 (100%) | 17,745,695 (94.77%) | 99.23% | 96.27% | 16,953,407 (47.77%) |
| Ac200-1 | Sample16-read1 | 17,657,891 (100%) | 16,698,100 (94.56%) | 99.32% | 96.87% | 15,982,028 (47.86%) |
|  | Sample16-read2 | 17,657,891 (100%) | 16,698,100 (94.56%) | 99.21% | 96.18% | 15,988,887 (47.88%) |
| Ac200-2 | Sample18-read1 | 18,389,213 (100%) | 17,447,657 (94.88%) | 99.37% | 97.09% | 16,714,607 (47.90%) |
|  | Sample18-read2 | 18,389,213 (100%) | 17,447,657 (94.88%) | 99.26% | 96.46% | 16,724,922 (47.93%) |

^a^ Q20 represents an error rate of less than 0.01.

^b^ Q30 represents an error rate of less than 0.001.

^c^ Number of high quality paired-end reads in each sample mapped to reference genome with the coverage in bracket.

Table S2. The cross-DEGs at more than two time points.

| Ntersection | Gene | log2(fold change (Ac/CK)) | | |
| --- | --- | --- | --- | --- |
|  |  | 45 min | 120 min | 200 min |
| 3 | SEO1 | -8.37 | -3.73 | -3.59 |
| 3 | HXT1 | -4.54 | -4.81 | -4.93 |
| 3 | PUT4 | -4.87 | -3.90 | -3.10 |
| 3 | ARG3 | -4.38 | -4.83 | -2.53 |
| 3 | ARG1 | -3.78 | -4.85 | -2.98 |
| 3 | PDC6 | -6.94 | -2.82 | -1.58 |
| 3 | SPO19 | -2.26 | -3.85 | -5.02 |
| 3 | INO1 | -1.45 | -3.37 | -6.31 |
| 3 | MET3 | -5.26 | -2.58 | -3.05 |
| 3 | SUL1 | -5.40 | -2.71 | -2.75 |
| 3 | MET6 | -4.37 | -3.04 | -3.40 |
| 3 | GSC2 | -3.64 | -3.44 | -3.66 |
| 3 | MET13 | -3.63 | -3.42 | -3.39 |
| 3 | STR3 | -5.15 | -2.95 | -1.72 |
| 3 | SUL2 | -4.28 | -2.76 | -2.75 |
| 3 | ITR1 | -2.37 | -3.05 | -3.90 |
| 3 | CLB1 | -2.00 | -3.22 | -4.01 |
| 3 | RPL7B | -1.68 | -3.55 | -3.91 |
| 3 | SPS1 | -3.11 | -3.33 | -2.56 |
| 3 | HSP33 | -3.23 | -2.22 | -3.44 |
| 3 | MET5 | -3.98 | -2.07 | -2.75 |
| 3 | SAM2 | -3.01 | -2.38 | -3.36 |
| 3 | MET10 | -4.18 | -2.26 | -2.25 |
| 3 | IDP2 | -3.24 | -2.73 | -2.55 |
| 3 | MEP2 | -3.04 | -3.21 | -2.27 |
| 3 | MET14 | -3.86 | -1.98 | -2.50 |
| 3 | RRT5 | -2.46 | -2.95 | -2.80 |
| 3 | HTD2 | -2.57 | -2.94 | -2.59 |
| 3 | ARG5,6 | -3.35 | -3.21 | -1.53 |
| 3 | RPS9A | -1.42 | -3.20 | -3.46 |
| 3 | MNN4 | -3.65 | -2.15 | -2.27 |
| 3 | RPL18B | -2.36 | -2.80 | -2.76 |
| 3 | GAS2 | -3.28 | -2.85 | -1.77 |
| 3 | RPL15A | -1.71 | -3.01 | -3.13 |
| 3 | MHT1 | -4.18 | -1.53 | -2.05 |
| 3 | MET17 | -3.59 | -1.91 | -2.14 |
| 3 | PMA1 | -1.74 | -2.93 | -2.95 |
| 3 | JLP1 | -2.58 | -2.47 | -2.43 |
| 3 | GAP1 | -2.94 | -2.37 | -2.15 |
| 3 | AGP1 | -3.59 | -1.15 | -2.70 |
| 3 | SPC25 | -2.74 | -2.23 | -2.41 |
| 3 | GTT3 | -2.45 | -2.54 | -2.31 |
| 3 | HAC1 | -1.91 | -2.57 | -2.81 |
| 3 | FMO1 | -3.48 | -1.68 | -1.92 |
| 3 | CTP1 | -1.49 | -2.86 | -2.71 |
| 3 | HSP12 | -3.80 | -1.98 | -1.26 |
| 3 | SAM3 | -3.27 | -1.79 | -1.95 |
| 3 | MMP1 | -4.14 | -1.34 | -1.52 |
| 3 | TRM11 | -1.58 | -2.50 | -2.90 |
| 3 | ATO3 | -2.00 | -2.92 | -1.99 |
| 3 | HES1 | -2.86 | -2.68 | -1.33 |
| 3 | PET10 | -2.68 | -2.28 | -1.88 |
| 3 | YPS1 | -2.29 | -2.14 | -2.41 |
| 3 | RPL22B | -1.76 | -2.01 | -3.07 |
| 3 | LYS21 | -1.32 | -3.13 | -2.37 |
| 3 | ODC2 | -1.91 | -2.74 | -2.15 |
| 3 | MET32 | -3.40 | -1.70 | -1.56 |
| 3 | RPS14B | -1.82 | -2.39 | -2.42 |
| 3 | GRE1 | -4.05 | -1.21 | -1.30 |
| 3 | SER33 | -3.51 | -1.58 | -1.47 |
| 3 | MEP1 | -1.55 | -2.58 | -2.41 |
| 3 | CHO2 | -2.17 | -2.04 | -2.32 |
| 3 | GIC1 | -2.51 | -2.15 | -1.86 |
| 3 | HNM1 | -1.95 | -2.22 | -2.35 |
| 3 | OPT1 | -3.15 | -1.62 | -1.74 |
| 3 | SFC1 | -2.00 | -2.11 | -2.40 |
| 3 | MET1 | -3.63 | -1.43 | -1.35 |
| 3 | BNA4 | -2.27 | -2.04 | -2.07 |
| 3 | HPT1 | -2.36 | -1.81 | -2.19 |
| 3 | RAD59 | -3.70 | -1.16 | -1.45 |
| 3 | GIT1 | -2.79 | -1.53 | -1.97 |
| 3 | PHM7 | -2.60 | -2.14 | -1.52 |
| 3 | ADE5,7 | -2.41 | -2.04 | -1.68 |
| 3 | GLT1 | -2.44 | -1.85 | -1.83 |
| 3 | SAM1 | -2.80 | -1.41 | -1.89 |
| 3 | AMA1 | -1.73 | -2.01 | -2.34 |
| 3 | PHM8 | -2.26 | -2.04 | -1.75 |
| 3 | ACC1 | -1.22 | -2.30 | -2.54 |
| 3 | FMP45 | -2.33 | -1.92 | -1.80 |
| 3 | GNP1 | -1.50 | -2.36 | -2.19 |
| 3 | MET2 | -2.75 | -1.50 | -1.77 |
| 3 | HVG1 | -2.26 | -2.52 | -1.25 |
| 3 | NIT1 | -2.77 | -2.21 | -1.03 |
| 3 | RMD6 | -3.17 | -1.47 | -1.35 |
| 3 | HBT1 | -3.27 | -1.39 | -1.32 |
| 3 | HIS4 | -2.27 | -2.51 | -1.16 |
| 3 | GLG2 | -2.40 | -1.87 | -1.62 |
| 3 | SCS3 | -2.04 | -1.80 | -2.02 |
| 3 | ALT2 | -2.64 | -1.65 | -1.56 |
| 3 | PMA2 | -2.26 | -2.42 | -1.14 |
| 3 | ELA1 | -2.20 | -1.95 | -1.62 |
| 3 | SYG1 | -2.86 | -1.19 | -1.72 |
| 3 | IPT1 | -1.74 | -2.01 | -2.00 |
| 3 | RPL3 | -1.43 | -2.03 | -2.27 |
| 3 | AGP3 | -4.94 | 1.12 | -1.88 |
| 3 | RTC2 | -1.79 | -2.68 | -1.21 |
| 3 | FLC1 | -1.87 | -1.71 | -2.09 |
| 3 | PDH1 | -2.33 | -1.33 | -1.99 |
| 3 | QDR3 | -1.76 | -2.45 | -1.43 |
| 3 | PIR1 | -1.20 | -2.34 | -2.09 |
| 3 | ARG8 | -1.72 | -2.81 | -1.09 |
| 3 | HSL7 | -1.87 | -1.80 | -1.95 |
| 3 | AGX1 | -1.89 | -1.36 | -2.32 |
| 3 | TKL2 | -2.79 | -1.21 | -1.56 |
| 3 | FUN26 | -1.58 | -1.97 | -2.01 |
| 3 | ATX1 | -1.85 | -1.91 | -1.77 |
| 3 | FAS1 | -1.03 | -2.13 | -2.27 |
| 3 | HBN1 | -2.79 | -1.40 | -1.24 |
| 3 | RPL31B | -1.12 | -2.04 | -2.24 |
| 3 | DIP5 | -2.68 | -1.49 | -1.21 |
| 3 | KRE6 | -1.84 | -1.68 | -1.78 |
| 3 | SHR5 | -2.19 | -1.45 | -1.61 |
| 3 | CPS1 | -2.53 | -1.61 | -1.07 |
| 3 | FAS2 | -1.14 | -2.05 | -2.02 |
| 3 | MUP1 | -2.45 | -1.44 | -1.30 |
| 3 | YAT1 | -2.12 | -1.16 | -1.89 |
| 3 | ALD3 | -2.59 | -1.35 | -1.24 |
| 3 | UPS2 | -1.16 | -1.88 | -2.13 |
| 3 | ISC1 | -1.99 | -1.61 | -1.53 |
| 3 | YGK3 | -1.82 | -1.99 | -1.29 |
| 3 | CAN1 | -1.97 | -1.83 | -1.23 |
| 3 | MTD1 | -2.45 | -1.54 | -1.02 |
| 3 | SPO74 | -1.56 | -1.62 | -1.83 |
| 3 | RPS7B | -1.11 | -1.72 | -2.17 |
| 3 | DAL3 | -2.31 | -1.48 | -1.19 |
| 3 | NCE103 | -1.83 | -1.96 | -1.18 |
| 3 | BNA2 | -1.87 | -1.70 | -1.37 |
| 3 | RMI1 | -2.03 | -1.12 | -1.78 |
| 3 | PRB1 | -2.28 | -1.37 | -1.26 |
| 3 | GDH1 | -1.17 | -1.71 | -2.02 |
| 3 | SNO1 | -1.31 | -2.28 | -1.27 |
| 3 | VPS62 | -1.71 | -1.83 | -1.32 |
| 3 | CDC13 | -2.04 | -1.42 | -1.39 |
| 3 | ARO1 | -1.35 | -1.96 | -1.53 |
| 3 | RPL4B | -1.27 | -1.76 | -1.81 |
| 3 | RPL1B | -1.07 | -1.55 | -2.20 |
| 3 | PEX21 | -1.45 | -1.95 | -1.42 |
| 3 | ECM13 | -1.66 | -1.65 | -1.47 |
| 3 | CTR2 | -1.98 | -1.36 | -1.41 |
| 3 | ECM3 | -1.58 | -1.56 | -1.58 |
| 3 | YIG1 | -1.82 | -1.53 | -1.36 |
| 3 | SPO75 | -2.11 | -1.19 | -1.36 |
| 3 | YCR062W | -2.00 | -1.51 | -1.15 |
| 3 | HOS1 | -2.03 | -1.57 | -1.01 |
| 3 | IML3 | -1.72 | -1.63 | -1.24 |
| 3 | PUS2 | -1.16 | -1.65 | -1.77 |
| 3 | PMT6 | -1.70 | -1.47 | -1.41 |
| 3 | GLK1 | -1.71 | -1.76 | -1.10 |
| 3 | DIA4 | -1.60 | -1.36 | -1.61 |
| 3 | GLO4 | -1.88 | -1.34 | -1.30 |
| 3 | MIG3 | -2.14 | -1.34 | -1.01 |
| 3 | PIR3 | -1.31 | -1.76 | -1.35 |
| 3 | SGF29 | -1.83 | -1.44 | -1.13 |
| 3 | KAP120 | -1.46 | -1.39 | -1.51 |
| 3 | YGP1 | -1.03 | -1.66 | -1.66 |
| 3 | DAL4 | -1.64 | -1.50 | -1.19 |
| 3 | GLY1 | -1.16 | -1.73 | -1.44 |
| 3 | GDT1 | -1.45 | -1.33 | -1.52 |
| 3 | IRC23 | -1.53 | -1.59 | -1.17 |
| 3 | UBC11 | -1.08 | -1.75 | -1.43 |
| 3 | MAK3 | -1.45 | -1.37 | -1.44 |
| 3 | SMP1 | -1.89 | -1.12 | -1.25 |
| 3 | DBF2 | -1.10 | -1.59 | -1.56 |
| 3 | BRR6 | -1.40 | -1.31 | -1.53 |
| 3 | LYP1 | -1.23 | -1.56 | -1.44 |
| 3 | PEX2 | -1.35 | -1.70 | -1.17 |
| 3 | YHK8 | -1.63 | -1.49 | -1.08 |
| 3 | ILV1 | -1.15 | -1.59 | -1.46 |
| 3 | MUD1 | -1.44 | -1.35 | -1.40 |
| 3 | ALP1 | -2.04 | -1.01 | -1.13 |
| 3 | YPS3 | -1.38 | -1.09 | -1.69 |
| 3 | YET2 | -1.02 | -1.07 | -2.07 |
| 3 | YOX1 | -1.84 | -1.03 | -1.26 |
| 3 | RTS2 | -1.15 | -1.53 | -1.41 |
| 3 | RTC1 | -1.45 | -1.31 | -1.30 |
| 3 | FOL1 | -1.39 | -1.15 | -1.49 |
| 3 | YKE4 | -1.68 | -1.23 | -1.11 |
| 3 | REC114 | -1.68 | -1.23 | -1.10 |
| 3 | SPO73 | -1.50 | -1.04 | -1.48 |
| 3 | LCB3 | -1.22 | -1.56 | -1.22 |
| 3 | YUR1 | -1.56 | -1.38 | -1.05 |
| 3 | SED1 | -1.00 | -1.65 | -1.34 |
| 3 | MIP6 | -1.40 | -1.42 | -1.14 |
| 3 | SET2 | -1.20 | -1.35 | -1.40 |
| 3 | PSP2 | -1.37 | -1.19 | -1.38 |
| 3 | DUS4 | -1.00 | -1.46 | -1.45 |
| 3 | ILV5 | -1.18 | -1.29 | -1.42 |
| 3 | SER3 | -1.62 | -1.20 | -1.07 |
| 3 | YEF1 | -1.56 | -1.10 | -1.22 |
| 3 | RAX1 | -1.29 | -1.20 | -1.39 |
| 3 | HUA2 | -1.66 | -1.13 | -1.07 |
| 3 | CAR2 | -1.60 | -1.08 | -1.18 |
| 3 | CNL1 | -1.47 | -1.02 | -1.35 |
| 3 | LOT5 | -1.14 | -1.33 | -1.35 |
| 3 | TOF2 | -1.25 | -1.17 | -1.39 |
| 3 | EPL1 | -1.36 | -1.25 | -1.20 |
| 3 | POX1 | -1.23 | -1.26 | -1.30 |
| 3 | PTC2 | -1.17 | -1.31 | -1.27 |
| 3 | BAG7 | -1.03 | -1.67 | -1.04 |
| 3 | ATG32 | -1.41 | -1.29 | -1.02 |
| 3 | MHO1 | -1.19 | -1.37 | -1.16 |
| 3 | CUE4 | -1.15 | -1.22 | -1.34 |
| 3 | EXO5 | -1.38 | -1.25 | -1.07 |
| 3 | PCA1 | -1.43 | -1.13 | -1.12 |
| 3 | RGS2 | -1.22 | -1.30 | -1.11 |
| 3 | CWH41 | -1.40 | -1.22 | -1.01 |
| 3 | INP53 | -1.38 | -1.04 | -1.19 |
| 3 | KSS1 | -1.02 | -1.12 | -1.45 |
| 3 | DCR2 | -1.31 | -1.27 | -1.01 |
| 3 | LEU3 | -1.00 | -1.37 | -1.20 |
| 3 | ROG1 | -1.24 | -1.12 | -1.20 |
| 3 | TCB3 | -1.34 | -1.01 | -1.20 |
| 3 | VMA1 | -1.18 | -1.15 | -1.21 |
| 3 | UPS3 | -1.12 | -1.23 | -1.17 |
| 3 | GBP2 | -1.18 | -1.17 | -1.15 |
| 3 | NSL1 | -1.25 | -1.12 | -1.06 |
| 3 | PPQ1 | -1.14 | -1.22 | -1.07 |
| 3 | POC4 | -1.13 | -1.23 | -1.06 |
| 3 | LDB17 | -1.27 | -1.03 | -1.07 |
| 3 | NAM8 | -1.02 | -1.15 | -1.19 |
| 3 | SNF6 | -1.15 | -1.02 | -1.19 |
| 3 | ALF1 | -1.09 | -1.17 | -1.07 |
| 3 | RER1 | -1.21 | -1.07 | -1.03 |
| 3 | NHA1 | -1.14 | -1.07 | -1.09 |
| 3 | SKG3 | -1.14 | -1.15 | -1.02 |
| 3 | PPH22 | -1.05 | -1.06 | -1.05 |
| 3 | ECM2 | 1.03 | 1.03 | 1.04 |
| 3 | SNC2 | 1.06 | 1.09 | 1.05 |
| 3 | RNH70 | 1.11 | 1.08 | 1.03 |
| 3 | YAF9 | 1.18 | 1.02 | 1.04 |
| 3 | APS3 | 1.19 | 1.02 | 1.06 |
| 3 | CDC31 | 1.22 | 1.03 | 1.02 |
| 3 | CRP1 | 1.26 | 1.02 | 1.03 |
| 3 | ESA1 | 1.23 | 1.08 | 1.02 |
| 3 | CWC24 | 1.10 | 1.17 | 1.05 |
| 3 | MIG1 | 1.03 | 1.16 | 1.15 |
| 3 | VPS30 | 1.10 | 1.24 | 1.00 |
| 3 | LCB5 | 1.30 | 1.05 | 1.01 |
| 3 | STP4 | 1.00 | 1.08 | 1.28 |
| 3 | MDM10 | 1.21 | 1.00 | 1.16 |
| 3 | TVP18 | 1.03 | 1.10 | 1.29 |
| 3 | PGA2 | 1.08 | 1.03 | 1.35 |
| 3 | GOS1 | 1.06 | 1.31 | 1.12 |
| 3 | LIN1 | 1.15 | 1.16 | 1.19 |
| 3 | PER33 | 1.19 | 1.17 | 1.15 |
| 3 | RAD10 | 1.11 | 1.25 | 1.17 |
| 3 | CPD1 | 1.17 | 1.18 | 1.19 |
| 3 | SSC1 | 1.48 | 1.02 | 1.06 |
| 3 | MGR1 | 1.04 | 1.02 | 1.53 |
| 3 | SPS4 | 1.01 | 1.22 | 1.39 |
| 3 | SNX41 | 1.00 | 1.19 | 1.46 |
| 3 | COA4 | 1.24 | 1.27 | 1.17 |
| 3 | RPT4 | 1.06 | 1.13 | 1.51 |
| 3 | NHP6A | 1.37 | 1.33 | 1.00 |
| 3 | APA1 | 1.62 | 1.07 | 1.08 |
| 3 | VPS51 | 1.04 | 1.15 | 1.59 |
| 3 | CMK2 | 1.09 | 1.37 | 1.33 |
| 3 | RTT103 | 1.31 | 1.25 | 1.23 |
| 3 | PEX3 | 1.28 | 1.23 | 1.30 |
| 3 | SGT2 | 1.30 | 1.15 | 1.38 |
| 3 | CYB5 | 1.40 | 1.40 | 1.02 |
| 3 | UFD1 | 1.01 | 1.40 | 1.43 |
| 3 | BUG1 | 1.18 | 1.26 | 1.43 |
| 3 | SRD1 | 1.03 | 1.28 | 1.58 |
| 3 | MVP1 | 1.20 | 1.35 | 1.34 |
| 3 | CDC27 | 1.31 | 1.43 | 1.18 |
| 3 | UMP1 | 1.13 | 1.23 | 1.56 |
| 3 | PTP2 | 1.13 | 1.43 | 1.37 |
| 3 | ELM1 | 1.42 | 1.30 | 1.22 |
| 3 | UBX3 | 1.04 | 1.47 | 1.44 |
| 3 | HSC82 | 1.74 | 1.13 | 1.09 |
| 3 | MCT1 | 1.33 | 1.14 | 1.48 |
| 3 | JSN1 | 1.17 | 1.46 | 1.37 |
| 3 | RPT5 | 1.04 | 1.34 | 1.64 |
| 3 | THI13 | 1.05 | 1.63 | 1.34 |
| 3 | FMP48 | 1.49 | 1.07 | 1.47 |
| 3 | SRN2 | 1.30 | 1.42 | 1.32 |
| 3 | YKE2 | 1.64 | 1.15 | 1.27 |
| 3 | BAT2 | 1.32 | 1.33 | 1.42 |
| 3 | UBX5 | 1.08 | 1.37 | 1.64 |
| 3 | RCN1 | 1.17 | 1.32 | 1.63 |
| 3 | MMM1 | 1.38 | 1.32 | 1.45 |
| 3 | CCC2 | 1.14 | 1.48 | 1.54 |
| 3 | FAL1 | 1.91 | 1.18 | 1.07 |
| 3 | HSP60 | 1.81 | 1.16 | 1.23 |
| 3 | RRG8 | 1.46 | 1.38 | 1.38 |
| 3 | PRM15 | 1.28 | 1.44 | 1.51 |
| 3 | DAD2 | 1.25 | 1.69 | 1.30 |
| 3 | SLZ1 | 1.15 | 1.43 | 1.68 |
| 3 | SRX1 | 1.55 | 1.18 | 1.56 |
| 3 | OAC1 | 1.98 | 1.31 | 1.03 |
| 3 | ICY1 | 1.43 | 1.35 | 1.57 |
| 3 | MRPL25 | 1.70 | 1.49 | 1.17 |
| 3 | RPN9 | 1.20 | 1.58 | 1.58 |
| 3 | HSP10 | 2.01 | 1.25 | 1.11 |
| 3 | SDS22 | 1.28 | 1.29 | 1.82 |
| 3 | SCL1 | 1.12 | 1.45 | 1.83 |
| 3 | HTB1 | 1.62 | 1.64 | 1.16 |
| 3 | RAD50 | 1.40 | 1.57 | 1.50 |
| 3 | RAM1 | 1.50 | 1.29 | 1.70 |
| 3 | HXK1 | 1.96 | 1.17 | 1.36 |
| 3 | EMI2 | 1.51 | 1.17 | 1.86 |
| 3 | SSE1 | 1.94 | 1.26 | 1.36 |
| 3 | HUL5 | 1.25 | 1.62 | 1.71 |
| 3 | BET4 | 1.33 | 1.66 | 1.63 |
| 3 | KAR4 | 1.48 | 1.55 | 1.63 |
| 3 | SWR1 | 1.35 | 1.88 | 1.44 |
| 3 | DAP1 | 1.41 | 1.53 | 1.74 |
| 3 | IRC10 | 1.12 | 1.31 | 2.27 |
| 3 | FLO11 | 1.33 | 1.44 | 1.95 |
| 3 | IME1 | 1.72 | 1.37 | 1.63 |
| 3 | COX17 | 2.12 | 1.53 | 1.10 |
| 3 | PGA3 | 1.46 | 1.72 | 1.61 |
| 3 | FCF1 | 2.10 | 1.31 | 1.40 |
| 3 | TMC1 | 1.36 | 1.68 | 1.78 |
| 3 | YPI1 | 1.39 | 1.58 | 1.87 |
| 3 | MGR3 | 1.57 | 1.70 | 1.57 |
| 3 | URA4 | 1.75 | 1.54 | 1.62 |
| 3 | PRM5 | 1.37 | 1.52 | 2.08 |
| 3 | AHC2 | 1.39 | 1.92 | 1.74 |
| 3 | REE1 | 1.98 | 1.54 | 1.58 |
| 3 | URA3 | 1.74 | 1.68 | 1.70 |
| 3 | HSP42 | 1.83 | 1.30 | 2.00 |
| 3 | HXT13 | 1.63 | 1.53 | 1.98 |
| 3 | SRL3 | 1.36 | 1.77 | 2.07 |
| 3 | FES1 | 2.14 | 1.26 | 1.90 |
| 3 | LEU2 | 1.82 | 1.67 | 1.82 |
| 3 | HSP78 | 2.20 | 1.10 | 2.02 |
| 3 | HSP104 | 2.17 | 1.29 | 1.86 |
| 3 | OPI10 | 1.92 | 1.41 | 2.12 |
| 3 | SLF1 | 1.87 | 2.07 | 1.54 |
| 3 | SNX4 | 1.56 | 1.90 | 2.04 |
| 3 | HEM13 | 1.64 | 1.90 | 1.97 |
| 3 | RCR1 | 1.51 | 1.61 | 2.49 |
| 3 | GSY1 | 1.69 | 1.83 | 2.14 |
| 3 | MDH2 | 1.59 | 2.00 | 2.30 |
| 3 | ECM11 | 1.58 | 1.88 | 2.45 |
| 3 | IZH4 | 1.77 | 1.85 | 2.30 |
| 3 | BIO2 | 1.91 | 2.03 | 2.02 |
| 3 | RIB4 | 1.88 | 2.02 | 2.14 |
| 3 | DDI1 | 1.73 | 1.98 | 2.39 |
| 3 | MGA1 | 1.73 | 2.12 | 2.27 |
| 3 | DAN1 | 1.32 | 2.12 | 2.71 |
| 3 | STI1 | 2.36 | 1.70 | 2.27 |
| 3 | HXT6 | 2.68 | 2.43 | 1.27 |
| 3 | PHD1 | 2.16 | 2.21 | 2.08 |
| 3 | SSA3 | 1.76 | 2.25 | 2.46 |
| 3 | AAC3 | 1.91 | 2.13 | 2.55 |
| 3 | URA1 | 2.44 | 2.31 | 1.90 |
| 3 | BTN2 | 2.57 | 1.62 | 2.56 |
| 3 | HXT7 | 2.15 | 2.42 | 2.29 |
| 3 | SSA4 | 2.14 | 2.08 | 2.64 |
| 3 | ZEO1 | 2.63 | 2.12 | 2.42 |
| 3 | LDS1 | 2.47 | 2.40 | 2.35 |
| 3 | DSF1 | 2.02 | 2.77 | 2.51 |
| 3 | TDA6 | 2.21 | 2.33 | 2.85 |
| 3 | ANB1 | 2.18 | 2.98 | 2.44 |
| 3 | HSP82 | 2.74 | 2.37 | 2.56 |
| 3 | TIR3 | 1.85 | 2.74 | 3.18 |
| 3 | PAU3 | 1.67 | 3.06 | 3.17 |
| 3 | CPR6 | 3.01 | 2.50 | 2.77 |
| 3 | CYC7 | 2.96 | 2.27 | 3.29 |
| 3 | PAU5 | 2.24 | 3.04 | 3.62 |
| 3 | TIP1 | 2.84 | 2.62 | 3.44 |
| 3 | PAU24 | 1.32 | 3.60 | 4.37 |
| 3 | HSP30 | 3.24 | 3.30 | 2.76 |
| 3 | TIR1 | 2.37 | 3.65 | 4.08 |
| 3 | CHA1 | 3.28 | 3.27 | 4.03 |
| 3 | GAL7 | 2.89 | 3.84 | 4.54 |
| 3 | RGI1 | 4.26 | 3.62 | 3.82 |
| 3 | PAU20 | 4.48 | 1.79769e+308 | 5.84 |
| 2 | YOL013W-B | -4.53 | -1.79769e+308 | -2.41 |
| 2 | LEE1 | -4.47 | -1.59 | -0.99 |
| 2 | ADE17 | -3.30 | -1.86 | -0.94 |
| 2 | BDS1 | -3.64 | -0.89 | -1.35 |
| 2 | LYS20 | -0.18 | -3.32 | -2.29 |
| 2 | NDE2 | -2.63 | -1.89 | -0.92 |
| 2 | RPL8A | -0.21 | -2.53 | -2.70 |
| 2 | LYS9 | 0.21 | -3.16 | -2.33 |
| 2 | RPL18A | -0.71 | -2.08 | -2.44 |
| 2 | MUM3 | -2.48 | -1.82 | -0.86 |
| 2 | SRY1 | -2.18 | -1.95 | -0.99 |
| 2 | SNZ1 | -1.85 | -2.35 | -0.84 |
| 2 | RPS3 | -0.87 | -1.76 | -2.26 |
| 2 | OPI3 | -0.86 | -1.70 | -2.32 |
| 2 | LYS2 | -0.94 | -2.38 | -1.56 |
| 2 | YSW1 | -2.00 | -1.95 | -0.91 |
| 2 | DUR3 | -2.32 | -1.74 | -0.77 |
| 2 | BNA3 | -2.80 | -1.04 | -0.98 |
| 2 | SHM2 | -2.70 | -1.17 | -0.93 |
| 2 | UTR5 | -2.19 | -1.30 | -1.24 |
| 2 | AQY2 | -0.47 | -1.77 | -2.43 |
| 2 | MIM2 | -0.98 | -1.76 | -1.92 |
| 2 | MET8 | -2.86 | -0.71 | -1.09 |
| 2 | MET16 | -2.71 | -1.03 | -0.92 |
| 2 | ADY3 | -2.57 | -1.20 | -0.88 |
| 2 | YRF1-4 | -1.78 | -1.49 | -1.30 |
| 2 | ATG36 | -1.89 | -1.68 | -0.98 |
| 2 | RPL2A | -0.75 | -1.56 | -2.19 |
| 2 | ARO4 | -0.63 | -2.13 | -1.74 |
| 2 | UGA4 | -2.04 | -1.72 | -0.67 |
| 2 | RPL26A | -0.77 | -1.58 | -2.06 |
| 2 | RPL23B | -0.78 | -1.68 | -1.95 |
| 2 | YHM2 | -0.85 | -1.52 | -2.01 |
| 2 | ALD5 | -0.86 | -1.86 | -1.63 |
| 2 | SPL2 | -2.14 | -1.68 | -0.52 |
| 2 | RRT6 | -1.91 | -1.43 | -0.98 |
| 2 | SPO13 | -1.54 | -1.81 | -0.96 |
| 2 | GDH3 | -2.24 | -1.27 | -0.78 |
| 2 | MCH4 | -1.88 | -1.67 | -0.73 |
| 2 | RPS24B | -0.85 | -1.42 | -1.94 |
| 2 | MRH1 | 0.40 | -2.08 | -2.50 |
| 2 | RPS16B | -0.75 | -1.53 | -1.90 |
| 2 | RPL16A | -0.82 | -1.38 | -1.97 |
| 2 | PPT2 | -1.98 | -1.29 | -0.86 |
| 2 | GND2 | -1.96 | -1.27 | -0.87 |
| 2 | EFT2 | -0.80 | -1.42 | -1.87 |
| 2 | SDT1 | -1.73 | -1.61 | -0.74 |
| 2 | CHO1 | -0.66 | -1.53 | -1.88 |
| 2 | FMP40 | -2.01 | -1.15 | -0.90 |
| 2 | PSD1 | -0.82 | -1.40 | -1.82 |
| 2 | RPS18B | -0.68 | -1.38 | -1.94 |
| 2 | RPL2B | -0.68 | -1.44 | -1.88 |
| 2 | RPL27B | -0.91 | -1.36 | -1.72 |
| 2 | ATR1 | -1.88 | -1.27 | -0.81 |
| 2 | RPL12A | -0.53 | -1.50 | -1.91 |
| 2 | BYE1 | -1.69 | -1.33 | -0.88 |
| 2 | HST3 | -0.87 | -1.46 | -1.52 |
| 2 | AAC1 | -1.55 | -1.35 | -0.91 |
| 2 | UTP22 | -0.90 | -1.16 | -1.74 |
| 2 | TPN1 | -1.68 | -1.23 | -0.87 |
| 2 | SHH4 | -1.78 | -1.27 | -0.71 |
| 2 | PAC2 | -1.45 | -1.36 | -0.95 |
| 2 | LYS4 | -0.13 | -1.97 | -1.64 |
| 2 | ALK1 | -0.61 | -1.38 | -1.74 |
| 2 | IDP1 | -0.81 | -1.85 | -1.08 |
| 2 | PET122 | -1.44 | -0.97 | -1.32 |
| 2 | SCW11 | -0.66 | -1.29 | -1.78 |
| 2 | PHO84 | -1.50 | -1.28 | -0.94 |
| 2 | FKS1 | -1.65 | -0.99 | -1.08 |
| 2 | MIG2 | -1.43 | -0.99 | -1.30 |
| 2 | ECM38 | -1.72 | -1.09 | -0.89 |
| 2 | ICT1 | -0.80 | -1.55 | -1.34 |
| 2 | RAD30 | -1.52 | -1.31 | -0.86 |
| 2 | CSH1 | -1.43 | -1.28 | -0.96 |
| 2 | RPS1B | -0.52 | -1.29 | -1.86 |
| 2 | MIR1 | -0.77 | -1.15 | -1.73 |
| 2 | GGC1 | -1.25 | -1.50 | -0.87 |
| 2 | AIM34 | -1.26 | -1.36 | -1.00 |
| 2 | RPS5 | -0.32 | -1.37 | -1.92 |
| 2 | RPL33B | -0.65 | -1.11 | -1.84 |
| 2 | SAH1 | -0.43 | -1.25 | -1.91 |
| 2 | BAP3 | -0.74 | -1.34 | -1.50 |
| 2 | RPS31 | -0.21 | -1.34 | -2.02 |
| 2 | YIP3 | -0.47 | -1.10 | -1.99 |
| 2 | PSA1 | -1.29 | -0.84 | -1.40 |
| 2 | AKR2 | -1.49 | -0.93 | -1.10 |
| 2 | CPA1 | -1.49 | -1.44 | -0.58 |
| 2 | ACO2 | -0.16 | -1.76 | -1.58 |
| 2 | LYS14 | -0.68 | -1.48 | -1.33 |
| 2 | RER2 | -0.75 | -1.24 | -1.50 |
| 2 | ALD6 | -0.62 | -1.47 | -1.38 |
| 2 | CDC26 | -0.93 | -1.53 | -1.01 |
| 2 | SMA2 | -1.42 | -0.95 | -1.11 |
| 2 | PKH1 | -1.27 | -1.28 | -0.92 |
| 2 | PCK1 | -1.33 | -0.87 | -1.26 |
| 2 | SRB2 | -0.78 | -1.30 | -1.37 |
| 2 | FCY2 | -1.09 | -0.99 | -1.37 |
| 2 | RPL14A | -0.52 | -1.26 | -1.65 |
| 2 | SIM1 | -1.27 | -0.99 | -1.18 |
| 2 | FMT1 | -1.41 | -0.84 | -1.19 |
| 2 | SIP1 | -1.30 | -1.17 | -0.96 |
| 2 | GTO3 | -1.31 | -1.53 | -0.58 |
| 2 | RAD51 | -1.52 | -0.82 | -1.06 |
| 2 | VTS1 | -0.92 | -1.02 | -1.46 |
| 2 | ENO1 | -0.98 | -1.14 | -1.27 |
| 2 | SWD1 | -1.48 | -0.88 | -1.02 |
| 2 | SIP2 | -1.37 | -1.22 | -0.79 |
| 2 | STE3 | -1.49 | -0.85 | -1.02 |
| 2 | FAR1 | -0.64 | -1.11 | -1.62 |
| 2 | PDC1 | 0.27 | -1.62 | -1.99 |
| 2 | SFH1 | -1.45 | -1.25 | -0.63 |
| 2 | EOS1 | -1.25 | -1.34 | -0.74 |
| 2 | ZRT2 | -1.42 | -0.80 | -1.10 |
| 2 | MOH1 | -1.41 | -1.01 | -0.89 |
| 2 | UBP10 | -0.77 | -1.07 | -1.46 |
| 2 | RPL24A | -0.17 | -1.32 | -1.80 |
| 2 | ADE12 | -1.46 | -1.24 | -0.59 |
| 2 | PGM1 | -1.17 | -1.00 | -1.12 |
| 2 | RPL19B | -0.41 | -1.23 | -1.63 |
| 2 | SPP2 | -1.06 | -1.34 | -0.87 |
| 2 | UBA3 | -1.47 | -1.08 | -0.71 |
| 2 | RPL6A | -0.42 | -1.29 | -1.54 |
| 2 | DFG5 | -1.31 | -1.05 | -0.89 |
| 2 | RPL27A | -0.46 | -1.08 | -1.70 |
| 2 | CIS3 | -0.80 | -1.03 | -1.42 |
| 2 | TYW3 | -0.75 | -1.16 | -1.33 |
| 2 | BET2 | -1.36 | -1.05 | -0.82 |
| 2 | YCH1 | -1.47 | -1.06 | -0.70 |
| 2 | HXT5 | -1.50 | -1.17 | -0.56 |
| 2 | MDS3 | -1.35 | -0.88 | -1.00 |
| 2 | GDH2 | -1.25 | -1.06 | -0.92 |
| 2 | UTP9 | -0.56 | -1.15 | -1.51 |
| 2 | RSP5 | -1.11 | -0.97 | -1.14 |
| 2 | TTI1 | -0.92 | -1.18 | -1.10 |
| 2 | BRL1 | -1.03 | -0.99 | -1.17 |
| 2 | RPL21A | -0.23 | -1.23 | -1.74 |
| 2 | IMO32 | -0.97 | -1.11 | -1.11 |
| 2 | PLB1 | -0.91 | -1.21 | -1.07 |
| 2 | CCW12 | -0.97 | -1.10 | -1.11 |
| 2 | DEF1 | -1.20 | -0.81 | -1.17 |
| 2 | RRT12 | -1.43 | -1.04 | -0.70 |
| 2 | RPL34A | -0.54 | -1.25 | -1.38 |
| 2 | DCS2 | -1.38 | -1.11 | -0.68 |
| 2 | ALG8 | -1.07 | -0.91 | -1.17 |
| 2 | RPS4A | -0.34 | -1.11 | -1.70 |
| 2 | ECM9 | -1.15 | -0.69 | -1.30 |
| 2 | RPS0A | -0.16 | -1.17 | -1.81 |
| 2 | RPL12B | -0.37 | -1.17 | -1.59 |
| 2 | CPA2 | -1.10 | -1.67 | -0.35 |
| 2 | RPS13 | -0.35 | -1.16 | -1.61 |
| 2 | RPS23A | -0.49 | -1.03 | -1.59 |
| 2 | ASI2 | -1.28 | -1.14 | -0.68 |
| 2 | HIP1 | -0.68 | -1.27 | -1.15 |
| 2 | KIP2 | -0.84 | -1.05 | -1.21 |
| 2 | RPL25 | -0.04 | -1.30 | -1.74 |
| 2 | GDS1 | -1.12 | -0.78 | -1.18 |
| 2 | DAL7 | -1.72 | -1.02 | -0.34 |
| 2 | SNT309 | -1.09 | -1.30 | -0.67 |
| 2 | SWI5 | 0.07 | -1.34 | -1.79 |
| 2 | ADH1 | -0.63 | -1.16 | -1.27 |
| 2 | RPS11A | -0.48 | -1.07 | -1.50 |
| 2 | ASC1 | 0.58 | -1.12 | -2.49 |
| 2 | ULA1 | -1.41 | -1.19 | -0.42 |
| 2 | ADA2 | -1.19 | -1.08 | -0.73 |
| 2 | KEX1 | -1.18 | -1.01 | -0.80 |
| 2 | RPS2 | -0.11 | -1.09 | -1.80 |
| 2 | PCL8 | -1.28 | -1.05 | -0.67 |
| 2 | IMD3 | -1.06 | -0.76 | -1.16 |
| 2 | YJL017W | -1.04 | -0.75 | -1.17 |
| 2 | FMP25 | -1.08 | -1.03 | -0.83 |
| 2 | EDS1 | -1.29 | -1.05 | -0.60 |
| 2 | RPS23B | -0.02 | -1.22 | -1.69 |
| 2 | RPL5 | -0.22 | -1.16 | -1.53 |
| 2 | EXO70 | -1.06 | -0.73 | -1.10 |
| 2 | RPL16B | -0.22 | -1.05 | -1.61 |
| 2 | MRM1 | -0.79 | -1.06 | -1.01 |
| 2 | RPS6B | -0.20 | -1.03 | -1.60 |
| 2 | DEG1 | -0.66 | -1.12 | -1.03 |
| 2 | TPO3 | -0.36 | -1.09 | -1.33 |
| 2 | ADO1 | -0.27 | -1.12 | -1.39 |
| 2 | RPS6A | -0.13 | -1.01 | -1.63 |
| 2 | RPL13B | -0.05 | -1.09 | -1.62 |
| 2 | SLC1 | -1.00 | -1.12 | -0.64 |
| 2 | RPL9A | 0.11 | -1.04 | -1.80 |
| 2 | LEU5 | -1.13 | -1.03 | -0.56 |
| 2 | PNS1 | -1.00 | -1.26 | -0.45 |
| 2 | RPS9B | 0.14 | -1.08 | -1.76 |
| 2 | ELP2 | -0.27 | -1.02 | -1.41 |
| 2 | LIA1 | -0.15 | -1.12 | -1.42 |
| 2 | LYS12 | 0.22 | -1.45 | -1.46 |
| 2 | SLX9 | -0.45 | -1.06 | -1.13 |
| 2 | CDC19 | -0.38 | -1.04 | -1.18 |
| 2 | NDT80 | -1.12 | -1.00 | -0.41 |
| 2 | RPL19A | -0.19 | -1.09 | -1.26 |
| 2 | GID8 | -1.30 | -1.03 | -0.20 |
| 2 | RPL15B | -1.09 | -1.11 | -0.32 |
| 2 | HIS5 | -1.04 | -1.33 | 0.00 |
| 2 | RPP2A | -0.05 | -1.01 | -1.25 |
| 2 | TAT1 | 0.72 | -1.30 | -1.61 |
| 2 | AIM20 | 0.38 | -1.15 | -1.36 |
| 2 | HSP31 | -1.04 | -1.10 | 0.10 |
| 2 | ESC8 | 0.20 | 1.02 | 1.12 |
| 2 | HXT11 | 0.23 | 1.06 | 1.10 |
| 2 | AAD6 | -0.24 | 1.20 | 1.46 |
| 2 | UBI4 | 0.03 | 1.07 | 1.36 |
| 2 | AAD16 | -0.23 | 1.17 | 1.53 |
| 2 | REX4 | 1.27 | 1.02 | 0.20 |
| 2 | NTF2 | 1.13 | 1.05 | 0.38 |
| 2 | HXT9 | -0.07 | 1.12 | 1.53 |
| 2 | SGS1 | 0.50 | 1.07 | 1.02 |
| 2 | RAD6 | 1.18 | 1.19 | 0.26 |
| 2 | MSA2 | 0.46 | 1.09 | 1.12 |
| 2 | FHN1 | 1.11 | 0.44 | 1.16 |
| 2 | TIR2 | 0.31 | 1.19 | 1.21 |
| 2 | PRE2 | 0.55 | 1.01 | 1.18 |
| 2 | FLO10 | 0.51 | 1.07 | 1.18 |
| 2 | ZPR1 | 1.09 | 0.44 | 1.24 |
| 2 | SOR1 | -0.01 | 1.18 | 1.60 |
| 2 | PTR2 | 0.20 | 1.33 | 1.26 |
| 2 | CMC2 | 1.19 | 1.12 | 0.51 |
| 2 | RIM15 | 0.52 | 1.18 | 1.11 |
| 2 | TSA1 | 0.69 | 1.08 | 1.07 |
| 2 | YIF1 | 0.64 | 1.07 | 1.15 |
| 2 | PRX1 | 0.58 | 1.08 | 1.22 |
| 2 | MGE1 | 1.26 | 1.08 | 0.53 |
| 2 | SNM1 | 1.08 | 1.12 | 0.70 |
| 2 | SWH1 | 1.03 | 1.03 | 0.85 |
| 2 | STB2 | 0.50 | 1.14 | 1.29 |
| 2 | RAD53 | 0.56 | 1.36 | 1.01 |
| 2 | PEX17 | 1.14 | 0.65 | 1.18 |
| 2 | IWR1 | 0.71 | 1.02 | 1.24 |
| 2 | ZIP1 | 0.60 | 1.11 | 1.27 |
| 2 | MSH5 | 0.47 | 1.14 | 1.37 |
| 2 | SPO20 | 0.50 | 1.07 | 1.42 |
| 2 | TDA4 | 0.58 | 1.03 | 1.37 |
| 2 | TRS23 | 1.02 | 0.81 | 1.16 |
| 2 | RAV2 | 1.03 | 0.89 | 1.08 |
| 2 | FYV10 | 0.62 | 1.01 | 1.37 |
| 2 | CDC37 | 0.81 | 1.17 | 1.03 |
| 2 | BUD13 | 0.85 | 1.15 | 1.01 |
| 2 | DRE2 | 0.99 | 1.03 | 1.00 |
| 2 | HMS1 | -0.34 | 1.55 | 1.81 |
| 2 | HRP1 | 0.87 | 1.13 | 1.05 |
| 2 | VPS15 | 1.02 | 1.16 | 0.87 |
| 2 | CDC53 | 0.79 | 1.08 | 1.18 |
| 2 | OXR1 | 0.76 | 1.00 | 1.29 |
| 2 | PRE5 | 0.77 | 1.07 | 1.23 |
| 2 | MVD1 | 0.88 | 1.03 | 1.15 |
| 2 | AIM32 | 1.10 | 1.12 | 0.85 |
| 2 | APC11 | 0.99 | 1.06 | 1.02 |
| 2 | CUZ1 | 0.76 | 1.01 | 1.31 |
| 2 | TAF11 | 1.02 | 0.99 | 1.06 |
| 2 | UBX4 | 0.76 | 1.01 | 1.30 |
| 2 | MAL11 | 0.48 | 1.10 | 1.50 |
| 2 | TRX2 | 0.54 | 1.23 | 1.32 |
| 2 | SSA2 | 1.29 | 0.76 | 1.05 |
| 2 | NFU1 | 0.94 | 1.13 | 1.04 |
| 2 | ATG33 | 0.72 | 1.02 | 1.38 |
| 2 | RSE1 | 1.01 | 1.09 | 1.01 |
| 2 | RPN12 | 0.72 | 1.15 | 1.27 |
| 2 | ELO1 | 0.59 | 1.01 | 1.55 |
| 2 | VTI1 | 0.84 | 1.17 | 1.15 |
| 2 | DIA1 | 1.18 | 1.05 | 0.93 |
| 2 | GEP4 | 0.90 | 1.26 | 1.00 |
| 2 | FIT2 | 0.82 | 1.03 | 1.32 |
| 2 | PRP8 | 1.11 | 1.20 | 0.88 |
| 2 | DMC1 | 0.71 | 1.08 | 1.40 |
| 2 | SBE2 | 0.93 | 1.10 | 1.18 |
| 2 | SBA1 | 0.90 | 1.07 | 1.24 |
| 2 | YDJ1 | 1.33 | 0.86 | 1.03 |
| 2 | CGI121 | 1.36 | 1.04 | 0.83 |
| 2 | PIM1 | 0.95 | 1.24 | 1.05 |
| 2 | RFA1 | 0.60 | 1.39 | 1.24 |
| 2 | PHO2 | 0.97 | 1.08 | 1.18 |
| 2 | REH1 | 0.67 | 1.04 | 1.53 |
| 2 | UBP6 | 0.81 | 1.07 | 1.37 |
| 2 | RCY1 | 0.78 | 1.26 | 1.21 |
| 2 | PAN2 | 0.93 | 1.31 | 1.05 |
| 2 | SOL1 | 1.09 | 1.18 | 1.01 |
| 2 | COX26 | 0.97 | 1.11 | 1.20 |
| 2 | PRM4 | 1.00 | 1.06 | 1.23 |
| 2 | PAA1 | 1.26 | 1.05 | 0.99 |
| 2 | RPN8 | 0.70 | 1.32 | 1.29 |
| 2 | CPR1 | 1.15 | 0.95 | 1.22 |
| 2 | THI11 | 0.75 | 1.44 | 1.14 |
| 2 | FAD1 | 0.84 | 1.26 | 1.23 |
| 2 | POL30 | 0.67 | 1.46 | 1.22 |
| 2 | FET4 | 1.51 | 1.00 | 0.83 |
| 2 | MRPL6 | 1.58 | 1.09 | 0.69 |
| 2 | HPA2 | 0.14 | 1.38 | 1.84 |
| 2 | PRE4 | 0.78 | 1.10 | 1.49 |
| 2 | HLJ1 | 0.94 | 1.27 | 1.17 |
| 2 | ATP11 | 1.42 | 1.20 | 0.78 |
| 2 | TDA11 | 0.77 | 1.33 | 1.30 |
| 2 | KTI12 | 1.69 | 1.12 | 0.60 |
| 2 | MRPL49 | 1.48 | 1.19 | 0.75 |
| 2 | SSK22 | 0.71 | 1.15 | 1.56 |
| 2 | IMA1 | 0.63 | 1.27 | 1.53 |
| 2 | EHD3 | 0.95 | 1.22 | 1.26 |
| 2 | PRE3 | 0.78 | 1.25 | 1.43 |
| 2 | GIR2 | 1.52 | 0.92 | 1.02 |
| 2 | PEX30 | 0.64 | 1.24 | 1.58 |
| 2 | COM2 | 1.40 | 1.21 | 0.89 |
| 2 | PEX10 | 0.91 | 1.21 | 1.38 |
| 2 | RNR4 | 1.22 | 1.31 | 0.97 |
| 2 | MCD1 | 0.53 | 1.60 | 1.37 |
| 2 | RHO4 | 1.00 | 1.23 | 1.28 |
| 2 | CIN5 | 1.00 | 1.16 | 1.36 |
| 2 | MND2 | 0.97 | 1.10 | 1.46 |
| 2 | MTR2 | 1.22 | 1.16 | 1.16 |
| 2 | PNC1 | 1.18 | 0.84 | 1.51 |
| 2 | RSM23 | 1.57 | 1.05 | 0.92 |
| 2 | PAU12 | 0.13 | 1.22 | 2.19 |
| 2 | ERO1 | 1.49 | 1.13 | 0.93 |
| 2 | RSM18 | 1.50 | 1.13 | 0.93 |
| 2 | RIX7 | 1.54 | 1.01 | 1.01 |
| 2 | RPN3 | 0.89 | 1.22 | 1.48 |
| 2 | SST2 | 0.79 | 1.30 | 1.52 |
| 2 | PAU23 | 0.53 | 1.35 | 1.72 |
| 2 | HXT10 | 0.41 | 1.11 | 2.09 |
| 2 | SHC1 | 0.63 | 1.24 | 1.75 |
| 2 | STE24 | 0.97 | 1.29 | 1.37 |
| 2 | YPS6 | 0.75 | 1.25 | 1.64 |
| 2 | PRE8 | 0.86 | 1.35 | 1.43 |
| 2 | EMC5 | 1.33 | 1.32 | 0.99 |
| 2 | NUR1 | 0.95 | 1.30 | 1.41 |
| 2 | ATG8 | 0.53 | 1.48 | 1.65 |
| 2 | BMT5 | 1.89 | 1.05 | 0.74 |
| 2 | ISF1 | 0.69 | 1.26 | 1.74 |
| 2 | MAL12 | 0.66 | 1.53 | 1.51 |
| 2 | RPT1 | 0.83 | 1.21 | 1.67 |
| 2 | SEM1 | 0.91 | 1.42 | 1.38 |
| 2 | COX7 | 1.54 | 1.60 | 0.59 |
| 2 | PRE1 | 0.92 | 1.38 | 1.44 |
| 2 | RPN5 | 0.95 | 1.37 | 1.43 |
| 2 | RDH54 | 0.87 | 1.45 | 1.44 |
| 2 | RPN6 | 0.98 | 1.29 | 1.57 |
| 2 | ERG28 | 0.98 | 1.57 | 1.40 |
| 2 | GLC3 | 0.97 | 1.22 | 1.80 |
| 2 | WSC4 | 1.60 | 1.26 | 1.26 |
| 2 | SUE1 | 0.57 | 1.34 | 2.22 |
| 2 | MAM1 | 0.95 | 1.62 | 1.76 |
| 2 | DOC1 | 0.99 | 1.51 | 1.92 |
| 2 | CMR3 | 0.83 | 1.59 | 2.04 |
| 2 | MTH1 | 1.28 | 1.67 | 1.67 |
| 2 | AGA1 | 1.00 | 1.74 | 2.01 |
| 2 | ECM29 | 0.79 | 1.93 | 2.06 |
| 2 | FUS1 | 0.94 | 1.73 | 2.25 |
| 2 | NDJ1 | 0.82 | 1.73 | 2.50 |
| 2 | MAG1 | 0.93 | 2.11 | 2.51 |
| 2 | PAU10 | 3.76 | 3.20 | 3.29 |

Table S3. Overrepresented functional categories of the cross-DEGs at more than two time points

| Pathway | No. of DEGs | Up-regulated genes | Down-regulated genes |
| --- | --- | --- | --- |
| H^+^ homeostasis | 11 | ***BTN2***, ***HSP30***, *RAV2* | ***GDT1***, *MIR1*, ***NCE103***, ***NHA1***, ***OPT1***, ***PMA1***, ***PMA2***, ***VMA1*** |
| NAD(P)/NAD(P)H homeostasis | 28 | *DRE2*, ***MDH2***, ***PGA2***, *PNC1* | *ADH1*, ***ALD3***, *ALD5*, *ALD6*, ***BNA2***, *BNA3*, ***BNA4***, ***FUN26***, ***GDH1***, *GDH2*, *GDH3*, ***GLT1***, *GND2*, *HST3*, *IDP1*, ***IDP2***, ***ILV5***, *LYS9*, ***MET13***, ***MTD1***, *NDE2*, *SDT1*, ***YEF1***, *YHM2* |
| Calcium homeostasis and signaling pathway | 10 | ***CDC31***, ***CMK2***, ***LCB5***, ***MDM10***, ***MMM1***, ***PTP2***, ***RCN1***, ***REE1*** | ***GDT1***, ***LCB3*** |
| Amino acid metabolism | 70 | ***BAT2***, ***CHA1***, *EHD3*, ***LEU2*** | *ACO2*, *ADE12*, ***AGX1***, ***ALD3***, ***ALT2***, ***ARG1***, ***ARG3***, ***ARG5****,6*, ***ARG8***, ***ARO1***, *ARO4*, ***BNA2***, *BNA3*, ***BNA4***, ***CAR2***, *CDC19*, *CPA1*, *CPA2*, *ECM38*, ***GDH1***, *GDH3*, *GLO4*, ***GLT1***, ***GLY1***, *GND2*, *GTO3*, ***GTT3***, ***HIS4***, *HIS5*, *IDP1*, ***IDP2***, ***ILV1***, ***ILV5***, ***LEU3***, *LYS12*, *LYS14*, *LYS2*, *LYS20*, ***LYS21***, *LYS4*, *LYS9*, ***MET1***, ***MET10***, ***MET13***, ***MET14***, *MET16*, ***MET17***, ***MET2***, ***MET3***, ***MET32***, ***MET5***, ***MET6***, *MET8*, ***MHT1***, ***ODC2***, *PDC1*, ***PDC6***, *SAH1*, ***SAM1***, ***SAM2***, ***SER3***, ***SER33***, *SHM2*, *SRY1*, ***STR3***, ***TKL2*** |
| Carbohydrate metabolism | 67 | *AAD16*, *AAD6*, ***DSF1***, ***EMI2***, ***FLO11***, ***GAL7***, *GLC3*, ***GSY1***, ***HXK1***, *IMA1*, *ISF1*, *MAL12*, ***MDH2***, ***MIG1***, *MTH1*, ***OAC1***, ***PRM15***, ***REE1***, *SOR1*, ***YPI1*** | *ACO2*, *ADH1*, ***ALD3***, *ALD5*, *ALD6*, *ALG8*, *CDC19*, ***CTP1***, ***CWH41***, *DAL7*, *ENO1*, *FKS1*, ***GAS2***, *GID8*, ***GLG2***, ***GLK1***, ***GLO4***, *GND2*, ***GSC2***, ***HSP12***, *HSP31*, ***HSP33***, *IDP1*, ***IDP2***, ***INO1***, ***KRE6***, *LEU5*, ***LYS21***, *MIG2*, ***MIG3***, *NDE2*, *PCK1*, *PDC1*, ***PDC6***, *PGM1*, *PSA1*, *SCW11*, ***SFC1***, *SHH4*, *SIM1*, *SIP1*, *SIP2*, ***TKL2***, ***YAT1***, *YHM2*, ***YIG1***, ***YUR1*** |
| Lipid metabolism | 33 | ***CYB5***, ***DAP1***, *ELO1*, *ERG28*, *GEP4*, ***LCB5***, ***MCT1***, *MVD1*, ***OPI10*** | ***ACC1***, *CHO1*, ***CHO2***, *CSH1*, ***FAS1***, ***FAS2***, ***HES1***, ***HTD2***, *ICT1*, ***INP53***, ***IPT1***, ***ISC1***, ***LCB3***, *MUM3*, *OPI3*, ***PET10***, *PLB1*, ***POX1***, *PPT2*, *PSD1*, ***ROG1***, ***SCS3***, *SLC1*, ***UPS2*** |
| Stress response | 35 | *AAD6*, *CIN5*, ***HSC82***, ***HSP104***, ***HSP30***, ***HSP42***, ***HSP60***, ***HSP78***, ***HSP82***, *OXR1*, *PRX1*, *RIM15*, ***SRX1***, *SSA2*, ***SSA3***, ***SSA4***, ***SSC1***, ***SSE1***, ***STI1***, ***TIP1***, *TIR2*, ***TMC1***, *TSA1*, *UBI4*, *WSC4*, *YDJ1*, *ZPR1* | *EOS1*, ***HSP12***, *HSP31*, ***HSP33***, ***MIG3***, ***PHM8***, *SIP1*, *SIP2* |
| Transcription regulation | 36 | *CGI121*, *CIN5*, ***ESA1***, *HMS1*, *KTI12*, ***MIG1***, *MSA2*, ***NHP6A***, ***PHD1***, *PHO2*, *RAD6*, ***RTT103***, *TAF11* | *ADA2*, *BYE1*, *EDS1*, *ELP2*, ***HAC1***, *HST3*, ***LEU3***, *LYS14*, ***MET32***, *MIG2*, ***MIG3***, *NDT80*, ***PPH22***, *RRT6*, ***SET2***, *SFH1*, ***SGF29***, ***SMP1***, ***SNF6***, *SRB2*, *SWI5*, ***YGK3***, ***YOX1*** |
| Histone modification | 17 | ***AHC2***, ***ESA1***, *HPA2*, ***HTB1***, ***SWR1***, ***UBX3***, ***YAF9*** | ***ACC1***, *ADA2*, *ALK1*, *BYE1*, ***EPL1***, ***HOS1***, *HST3*, ***SET2***, ***SGF29***, *SWD1* |
| Transporters | 35 | *FET4*, *HXT10*, *HXT11*, ***HXT13***, ***HXT6***, ***HXT7***, *HXT9*, *MAL11*, ***OAC1***, *PTR2* | ***ALP1***, ***ATO3***, ***ATX1***, ***CTP1***, ***CTR2***, *DUR3*, ***FLC1***, ***FUN26***, *GGC1*, ***HNM1***, ***HXT1***, *HXT5*, ***ITR1***, ***NHA1***, ***ODC2***, ***OPT1***, *PHO84*, ***QDR3***, ***RTC2***, ***SFC1***, *TAT1*, *TPO3*, ***YHK8***, ***YKE4***, *ZRT2* |
| Permeases | 24 | *Null* | ***AGP1***, ***AGP3***, *BAP3*, ***CAN1***, ***DAL4***, ***DIP5***, *FCY2*, ***GAP1***, ***GIT1***, ***GNP1***, *HIP1*, ***LYP1***, *MCH4*, ***MEP1***, ***MEP2***, ***MMP1***, ***MUP1***, ***PUT4***, ***SAM3***, ***SEO1***, ***SUL1***, ***SUL2***, *TPN1*, *UGA4* |
| Protein folding and stabilization | 26 | *CDC37*, *CPR1*, ***CPR6***, *EMC5*, *ERO1*, *HLJ1*, ***HSC82***, ***HSP10***, ***HSP104***, ***HSP42***, ***HSP60***, ***HSP78***, ***HSP82***, *MGE1*, *SBA1*, ***SGT2***, *SSA2*, ***SSA3***, ***SSA4***, ***SSC1***, ***SSE1***, ***STI1***, *TSA1*, *YDJ1*, ***YKE2*** | ***FMO1*** |
| Ubiquitin-dependent protein catabolic process | 47 | *APC11*, ***CDC27***, ***CDC31***, *CDC53*, *CUZ1*, ***DDI1***, *DOC1*, ***FES1***, *FYV10*, *HLJ1*, ***HUL5***, *MND2*, *PEX10*, *PRE1*, *PRE2*, *PRE3*, *PRE4*, *PRE5*, *PRE8*, *RAD6*, *RPN12*, *RPN3*, *RPN5*, *RPN6*, *RPN8*, ***RPN9***, *RPT1*, ***RPT4***, ***RPT5***, ***SCL1***, *SEM1*, ***SRN2***, *UBI4*, *UBP6*, ***UBX3***, *UBX4*, ***UBX5***, ***UFD1***, ***UMP1***, *YDJ1* | *ASI2*, *CDC26*, *DEF1*, ***ELA1***, *RSP5*, ***UBC11***, *UBP10* |
| Vesicle-mediated transport | 23 | ***APS3***, *ATG8*, ***BET4***, ***BTN2***, ***BUG1***, ***DDI1***, ***GOS1***, ***MVP1***, ***RCR1***, *RCY1*, ***SNC2***, ***SNX4***, ***SNX41***, *TRS23*, *TRX2*, ***TVP18***, *VPS15*, ***VPS30***, ***VPS51***, *VTI1*, *YIF1* | *BET2*, *YIP3* |
| Protein synthesis | 70 | ***ANB1***, ***FAL1***, ***FCF1***, ***FES1***, *MTR2*, *REH1*, *REX4*, *RIX7*, ***RNH70***, *SNM1* | *ASC1*, ***DIA4***, *EFT2*, *FMT1*, *PET122*, *RPL12A*, *RPL12B*, *RPL13B*, *RPL14A*, ***RPL15A***, *RPL15B*, *RPL16A*, *RPL16B*, *RPL18A*, ***RPL18B***, *RPL19A*, *RPL19B*, ***RPL1B***, *RPL21A*, ***RPL22B***, *RPL23B*, *RPL24A*, *RPL25*, *RPL26A*, *RPL27A*, *RPL27B*, *RPL2A*, *RPL2B*, ***RPL3***, ***RPL31B***, *RPL33B*, *RPL34A*, ***RPL4B***, *RPL5*, *RPL6A*, ***RPL7B***, *RPL8A*, *RPL9A*, *RPP2A*, *RPS0A*, *RPS11A*, *RPS13*, ***RPS14B***, *RPS16B*, *RPS18B*, *RPS1B*, *RPS2*, *RPS23A*, *RPS23B*, *RPS24B*, *RPS3*, *RPS31*, *RPS4A*, *RPS5*, *RPS6A*, *RPS6B*, ***RPS7B***, ***RPS9A***, *RPS9B*, *UTP22* |
| MAPK signaling pathway | 18 | *CDC37*, ***FLO11***, *FUS1*, ***PTP2***, *SSK22*, ***ZEO1*** | ***AMA1***, *FAR1*, *FKS1*, ***GSC2***, ***KSS1***, *PKH1*, ***PPQ1***, ***PTC2***, ***SMP1***, ***SPS1***, *STE3*, ***YPS1*** |
| Filamentous growth | 16 | *DIA1*, *FLO10*, ***FLO11***, *HMS1*, ***ICY1***, ***MGA1***, ***MIG1***, ***PHD1*** | *DFG5*, ***DIA4***, ***GAP1***, ***KSS1***, ***MEP2***, ***MHO1***, *MIG2*, ***YPS1*** |
| Pheromone response | 10 | *FUS1*, ***KAR4***, *PRM4*, *SST2*, *STE24* | *FAR1*, *KEX1*, ***KSS1***, ***PPQ1***, *STE3* |
| Cell wall organization | 28 | ***RCR1***, *SBE2*, ***TIP1***, *YPS6* | *CCW12*, *CIS3*, ***CWH41***, *DFG5*, ***ECM13***, ***ECM3***, *ECM9*, *FKS1*, ***FLC1***, ***FMP45***, ***GAS2***, ***GSC2***, ***KRE6***, ***PIR1***, ***PIR3***, *PKH1*, *PSA1*, *SCW11*, ***SED1***, *SIM1*, ***YGP1***, ***YPS1***, ***YPS3***, ***YUR1*** |
| Ascospore wall assembly | 12 | ***LDS1*** | *ADY3*, ***AMA1***, ***GAS2***, ***GSC2***, *MUM3*, ***QDR3***, *RRT12*, *SMA2*, ***SPO73***, ***SPO75***, ***SPS1*** |
| Ascospore formation | 23 | ***EMI2***, ***LDS1***, *SHC1*, ***SLZ1***, *SPO20* | *ADY3*, ***AMA1***, ***FMP45***, ***GAS2***, ***GSC2***, ***IML3***, *MUM3*, *NDT80*, ***QDR3***, ***RMD6***, *RRT12*, ***RRT5***, *SMA2*, ***SPO19***, ***SPO73***, ***SPO74***, ***SPO75***, ***SPS1*** |
| Cell cycle | 65 | *APC11*, ***CDC27***, ***CDC31***, *CDC37*, *CDC53*, ***DAD2***, *DMC1*, *DOC1*, ***ECM11***, ***EMI2***, ***IME1***, ***KAR4***, ***LDS1***, *MAM1*, *MCD1*, *MND2*, *MSA2*, *MSH5*, *NDJ1*, *NUR1*, ***RAD50***, *RAD53*, *RDH54*, *RFA1*, *RIM15*, *SHC1*, ***SLZ1***, *SPO20*, *ZIP1* | *ADY3*, *ALK1*, ***AMA1***, *CDC26*, ***CLB1***, ***DBF2***, ***DCR2***, *FAR1*, ***FMP45***, ***GAS2***, ***GSC2***, ***HSL7***, ***IML3***, *KIP2*, *MDS3*, *MUM3*, ***NAM8***, *NDT80*, ***PPH22***, ***QDR3***, ***REC114***, ***RMD6***, ***RMI1***, *RRT12*, ***RRT5***, *SFH1*, *SMA2*, *SPO13*, ***SPO19***, ***SPO73***, ***SPO74***, ***SPO75***, ***SPS1***, *SWI5*, ***YOX1***, *YSW1* |
| Mitotic cell cycle | 29 | *APC11*, ***CDC27***, ***CDC31***, *CDC37*, *CDC53*, ***DAD2***, *DMC1*, *DOC1*, *MCD1*, *MND2*, *MSA2*, *NUR1*, *RAD53*, *RFA1* | *ALK1*, *CDC26*, ***CLB1***, ***DBF2***, ***DCR2***, *FAR1*, ***HSL7***, ***IML3***, *KIP2*, *NDT80*, ***PPH22***, ***RMI1***, *SFH1*, *SWI5*, ***YOX1*** |
| Meiotic cell cycle | 48 | *APC11*, ***CDC27***, *DMC1*, *DOC1*, ***ECM11***, ***EMI2***, ***IME1***, ***KAR4***, ***LDS1***, *MAM1*, *MND2*, *MSH5*, *NDJ1*, ***RAD50***, *RDH54*, *RFA1*, *RIM15*, *SHC1*, ***SLZ1***, *SPO20*, *ZIP1* | *ADY3*, ***AMA1***, *CDC26*, ***CLB1***, *FAR1*, ***GAS2***, ***GSC2***, ***IML3***, *MDS3*, *MUM3*, ***NAM8***, *NDT80*, ***PPH22***, ***QDR3***, ***REC114***, ***RMD6***, ***RMI1***, *RRT12*, ***RRT5***, *SMA2*, *SPO13*, ***SPO19***, ***SPO73***, ***SPO74***, ***SPO75***, ***SPS1***, *YSW1* |
| DNA repair | 20 | *DMC1*, ***ESA1***, ***HTB1***, *MAG1*, *MCD1*, *MSH5*, ***NHP6A***, *PAN2*, *POL30*, ***RAD10***, ***RAD50***, *RAD53*, *RAD6*, *RDH54*, *RFA1*, *SGS1* | *DEF1*, *RAD30*, *RAD51*, ***RAD59*** |
| Programmed cell death | 22 | ***AAC3***, *ATG8*, *ATG33*, ***CYC7***, *DRE2*, ***ESA1***, *FYV10*, ***HTB1***, *MCD1*, ***MDM10***, ***MMM1***, ***SNX4***, *VPS15*, ***VPS30*** | *AAC1*, ***ATG32***, *ATG36*, ***CDC13***, ***EPL1***, ***ISC1***, *KEX1*, ***SNO1*** |
| Apoptosis | 11 | ***AAC3***, ***CYC7***, *DRE2*, *FYV10*, ***HTB1***, *MCD1* | *AAC1*, ***CDC13***, ***ISC1***, *KEX1*, ***SNO1*** |
| Autophagy | 9 | *ATG8*, *ATG33*, ***ESA1***, *VPS15*, ***VPS30*** | ***ATG32***, *ATG36*, ***CDC13***, ***EPL1*** |
| Mitophagy | 6 | *ATG8*, *ATG33*, ***MDM10***, ***MMM1***, ***SNX4*** | ***ATG32*** |

The common DEGs at three time points were emphasized in bold.

Table S4. MIPS functional categories of DEGs in different time during acetic acid treatment.

| Functional category^a^ | No. of genes | Gene match (genome match)^b^ | *P* value |
| --- | --- | --- | --- |
| Up-regulated at 45 min (374 out of 381 genes are found) | | | |
| 16 Protein with binding function or cofactor requirement (structural or catalytic) | 182 | 48.6/41.4 | 2.14E-03 |
| 11 Transcription | 114 | 30.4/20.8 | 3.35E-06 |
| 32 Cell rescue, defense and virulence | 79 | 21.1/15.4 | 1.69E-03 |
| 12 Protein synthesis | 77 | 20.5/9.24 | 3.23E-12 |
| Up-regulated at 120 min (300 out of 307 genes are found) | | | |
| 16 Protein with binding function or cofactor requirement (structural or catalytic) | 140 | 46.6/41.4 | 3.46E-02 |
| 14 Protein fate (folding, modification, destination) | 104 | 34.6/23.9 | 1.21E-05 |
| 32 Cell rescue, defense and virulence | 89 | 29.6/15.4 | 1.30E-10 |
| 10 Cell cycle and DNA processing | 83 | 27.6/22.5 | 1.96E-02 |
| Up-regulated at 200 min (371 out of 377 genes are found) | | | |
| 16 Protein with binding function or cofactor requirement (structural or catalytic) | 176 | 47.4/41.4 | 9.27E-03 |
| 14 Protein fate (folding, modification, destination) | 137 | 36.9/23.9 | 5.45E-09 |
| 32 Cell rescue, defense and virulence | 113 | 30.4/15.4 | 4.08E-14 |
| 18 Regulation of metabolism and protein function | 50 | 13.4/9.22 | 3.46E-03 |
| Down-regulated at 45 min (507 out of 512 genes are found) | | | |
| 01 Metabolism | 259 | 51.0/33.4 | 8.87E-18 |
| 20 Cellular transport, transport facilities and transport routes | 138 | 27.2/22.9 | 1.00E-02 |
| 43 Cell type differentiation | 64 | 12.6/9.88 | 2.12E-02 |
| 34 Interaction with the environment | 61 | 12.0/9.07 | 1.16E-02 |
| 02 Energy | 55 | 10.8/8.11 | 1.37E-02 |
| Down-regulated at 120 min (448 out of 451 genes are found) | | | |
| 01 Metabolism | 217 | 48.4/33.4 | 6.76E-12 |
| 20 Cellular transport, transport facilities and transport routes | 86 | 19.1/15.3 | 1.26E-02 |
| 12 Protein synthesis | 66 | 14.7/9.24 | 6.13E-05 |
| Down-regulated at 200 min (494 out of 497 genes are found) | | | |
| 16 Protein with binding function or cofactor requirement (structural or catalytic) | 255 | 51.6/41.4 | 1.23E-06 |
| 01 Metabolism | 198 | 40/33.4 | 7.78E-04 |
| 12 Protein synthesis | 142 | 28.7/9.24 | 1.13E-39 |

^a^ Functional categories are overrepresented with *P* value <0.05 and gene match >10%.

^b^ The percent value of genes in all DEGs and the whole yeast genome that match in corresponding functional category.

Table S6. *S. cerevisiae* strains constructed and used in this study.

| Strains | Genotype | Source |
| --- | --- | --- |
| W303-1B | MATα ura3-1 trp1-1 leu2-3,112 his3-11,15 ade2-1 can1-100 | EUROSCARF |
| W303 mtGFP | W303-1B harboring pYX232-mtGFP | This study |
| W303 pHluorin | W303-1B harboring pYES2-ACT1-pHluorin | This study |
| W303 mtpHluorin | W303-1B harboring pYES2-ACT1-mtpHluorin | This study |
| W303 pESC-ura | W303-1B harboring pESC-ura | This study |
| W303 pESC-*ADA2* | W303-1B harboring pESC-*ADA2* | This study |
| W303 pESC-*AHC2* | W303-1B harboring pESC-*AHC2* | This study |
| W303 pESC-*ESA1* | W303-1B harboring pESC-*ESA1* | This study |
| W303 pESC-*EPL1* | W303-1B harboring pESC-*EPL1* | This study |
| W303 pESC-*HPA2* | W303-1B harboring pESC-*HPA2* | This study |
| W303 pESC-*HOS1* | W303-1B harboring pESC-*HOS1* | This study |
| W303 pESC-*HST3* | W303-1B harboring pESC-*HST3* | This study |
| W303 pESC-*SGF29* | W303-1B harboring pESC-*SGF29* | This study |
| W303 pESC-*YAF9* | W303-1B harboring pESC-*YAF9* | This study |
| W303 pESC-*SET2* | W303-1B harboring pESC-*SET2* | This study |
| BY4742 | MATα his3Δ1 leu2Δ0 lys2Δ0 ura3Δ0 | EUROSCARF |
| *ADA2*Δ | BY4742 *ADA2*::KanMX4 | EUROSCARF |
| *AHC2*Δ | BY4742 *AHC2*::KanMX4 | EUROSCARF |
| *HPA2*Δ | BY4742 *HPA2*::KanMX4 | EUROSCARF |
| *HOS1*Δ | BY4742 *HOS1*::KanMX4 | EUROSCARF |
| *HST3*Δ | BY4742 *HST3*::KanMX4 | EUROSCARF |
| *SGF29*Δ | BY4742 *SGF29*::KanMX4 | EUROSCARF |
| *YAF9*Δ | BY4742 *YAF9*::KanMX4 | EUROSCARF |
| BY4743 | MATa/MATα his3Δ1/his3Δ1 leu2Δ0/leu2Δ0 met15Δ0/MET15 LYS2/lys2Δ0 ura3Δ0/ura3Δ0 | EUROSCARF |
| *esa1*Δ/*ESA1* | BY4743 *esa1*::kanMX4/*ESA1* | EUROSCARF |
| *epl1*Δ/*EPL1* | BY4743 *epl1*::kanMX4/*EPL1* | EUROSCARF |

**Table S7**. Primers and restriction enzymes for the recombinant plasmids used in this study.

| Plasmid | Primer | Restriction enzyme |
| --- | --- | --- |
| pESC-ADA2 | AACCCTCACTAAAGGGCGGCCGCATGTCAAACAAGTTTCACTGTGAC (forward)  TTGTAATCCATCGATACTAGTTTACATCCAATTCTGGCTCTGG (reverse) | Not I  Spe I |
| pESC-AHC2 | AACCCTCACTAAAGGGCGGCCGCATGATCACCCCAAAGGGA (forward)  TTGTAATCCATCGATACTAGTTCATAATAAGCCATCTTCATACAA (reverse) | Not I  Spe I |
| pESC-ESA1 | AACCCTCACTAAAGGGCGGCCGCATGTCCCATGACGGAAAAG (forward)  TTGTAATCCATCGATACTAGTTTACCAGGCAAAGCGTAAC (reverse) | Not I  Spe I |
| pESC-EPL1 | AACCCTCACTAAAGGGCGGCCGCATGCCGACACCTTCAAAC (forward)  TTGTAATCCATCGATACTAGTTCATGATGAATTTTTCTGGGTT (reverse) | Not I  Spe I |
| pESC-HPA2 | AACCCTCACTAAAGGGCGGCCGCATGTCCAACACTAGCGAAG (forward)  TTGTAATCCATCGATACTAGTTTAATATCCCTTCCTCTTGTATA (reverse) | Not I  Spe I |
| pESC-HOS1 | AACCCTCACTAAAGGGCGGCCGCATGTCGAAATTGGTCATATCA (forward)  TTGTAATCCATCGATACTAGTTTACAGTTCGTAAAACTTCATAAG (reverse) | Not I  Spe I |
| pESC-HST3 | AACCCTCACTAAAGGGCGGCCGCATGACTTCAGTATCGCCCTC (forward)  (TTGTAATCCATCGATACTAGTTTATGAGGCTTGGTTGTCACC (reverse) | Not I  Spe I |
| pESC-SGF29 | AACCCTCACTAAAGGGCGGCCGCATGGACGGATATTGGGATGTTG (forward)  TTGTAATCCATCGATACTAGTCTATTTCCTTGCTAGGTTTGCC (reverse) | Not I  Spe I |
| pESC-YAF9 | AACCCTCACTAAAGGGCGGCCGCATGGCTCCGACAATAAGCAA (forward)  TTGTAATCCATCGATACTAGTCTAACTTCCGTTAATGGCTTCTT (reverse) | Not I  Spe I |
| pESC-SET2 | AACCCTCACTAAAGGGCGGCCGCATGTCGAAGAACCAAAGTGTGAG (forward)  TTGTAATCCATCGATACTAGTTTATGATGATGTTGAAGGTGGAGGA (reverse) | Not I  Spe I |

**Table S8. Primers used in qPCR analysis**

| Gene | Forward primer (5' → 3') | Reverse primer (5' → 3') | Length (bp) |
| --- | --- | --- | --- |
| ACT1 | TCCCAGGTATTGCCGAAAGAATGC | GCCAAGATAGAACCACCAATCCAGA | 124 |
| ESA1 | TGGTCGGACACTCTCATAACGCTAT | GCTGACCCTTGTAATATCGCAGGAT | 145 |
| BTN2 | TTCCGAAGGTGGCATCAACGAAC | CGCTTTCTCCGCTTCTTCCTCCT | 115 |
| HSP30 | ATGCTACGGACGATGTGGAAGATG | GCTTGCTCTGCTTCAGGTTCGG | 115 |
| RGI1 | CCCACCATTTGTGCTACACGAGTC | TCAGGTTTCCTAACGGCTTGCTTAA | 150 |
| ZEO1 | GCTACTCCAGAAGCTGAACAGGTGA | TTGGTTTCCTTCTTCTCACTGACGG | 143 |
| EPL1 | GCATCCTCTTCTTCAACATCACAGC | AGAGCATCCACGTCTTCCAATACAA | 101 |
| VMA1 | GCTACCTACCAGACTTACGCTCCAA | TGTCAGACAATCCATCACCAATCCA | 148 |
| GSC2 | TTGCTGACCCGTGAGTTCAAGAATG | CCTTGTCGGTTGAGTCCATGCCATA | 105 |
| PTP2 | TCCGCATAATGGTGACCTTACTTCC | TCTACGCCGCATGAATCTAACCAAT | 102 |
| ATG8 | AAGGCGGAGTCGGAGAGGATTG | CCTACGGTAAGGTCAGCAGGAACT | 131 |
| ATG32 | CACGATTCCGCAACATTCCC | TTGCCCAGGCTGGCAGATC | 139 |
| RSP5 | CCTGGGATGACCCAAGACT | CGGGCTGGGACCTGAAAT | 99 |
| ACC1 | TTCCACCTCAGTTAATTCGCCGTTT | TTATTCCGCCAAGCATCGTCATCC | 123 |
| TIP1 | GGATGGAGCGGCAGAGGATGTA | CTGCTGCTCTTGCCTCCGTTTC | 116 |
| FES1 | CTCGTCAGACAAGCACTCAATCGTA | TGAGAGCCATTGCCTTATTGACCG | 119 |

**Supplementary Figures**

**Fig. S1 *In situ* calibration curves of pH_cyt_ and pH_mit_.** Ratio of emission intensity at 512 nm upon excitation at 390 nm and 470 nm (R_390/470_) were plotted against pH after subtracting background for pH_cyt_ (A) and pH_mit_ (B).


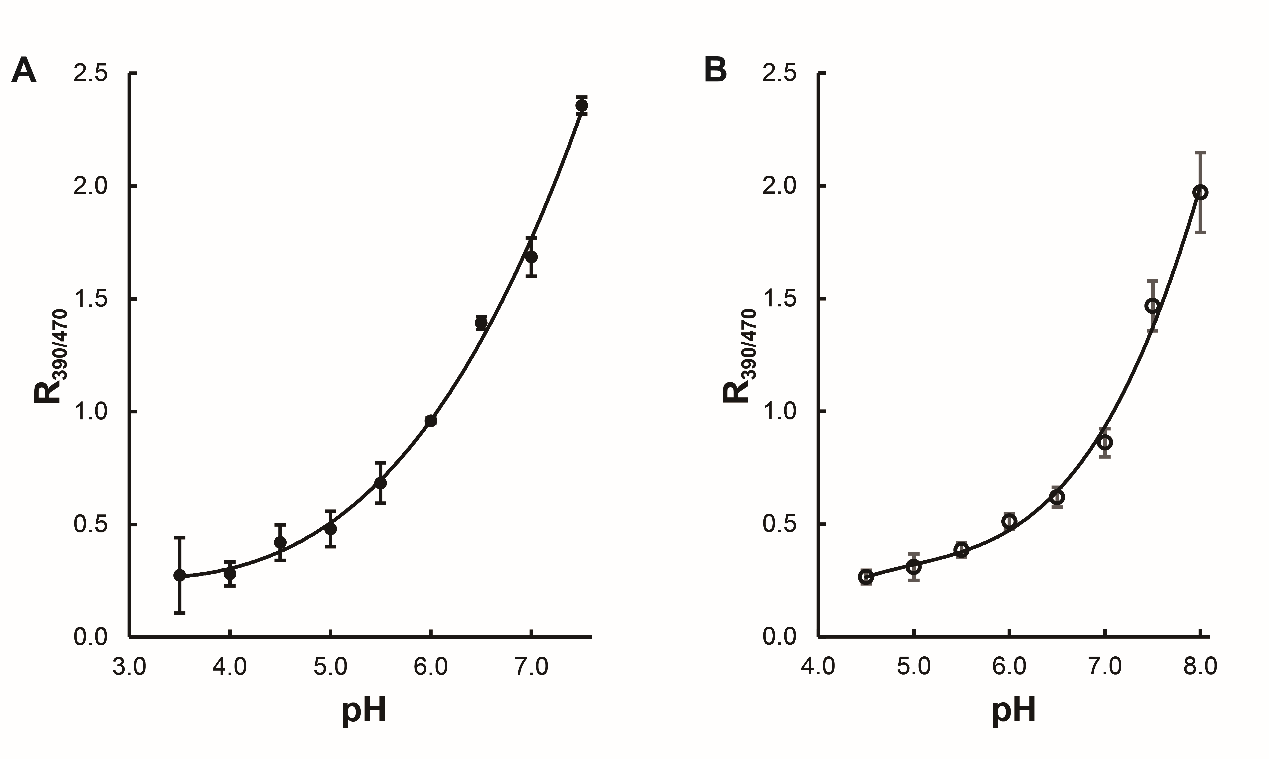


**Fig. S2 Validation of qPCR.** Histogram (A) and correlation analysis (B) for comparison of RNA-seq and qPCR data. The fold changed means the ratio of mRNA level of genes in acetic acid group against the control group. *ACT1* serves as a reference in qPCR.


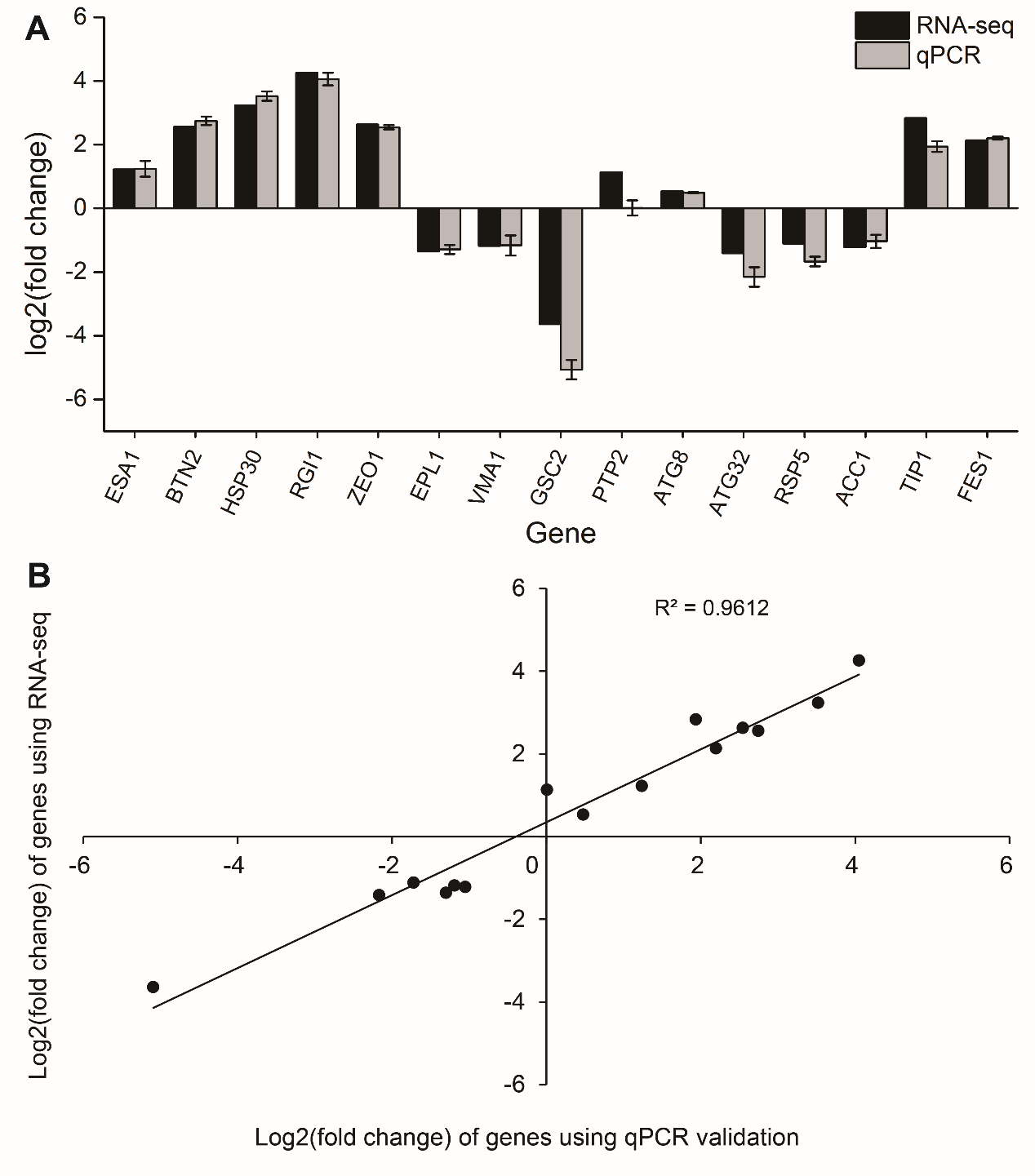


**Fig. S3 Gene ontology classification of DEGs at three time points.** The DEGs were classified into 3 functional categories: biological process (A), cellular component (B) and molecular function (C).


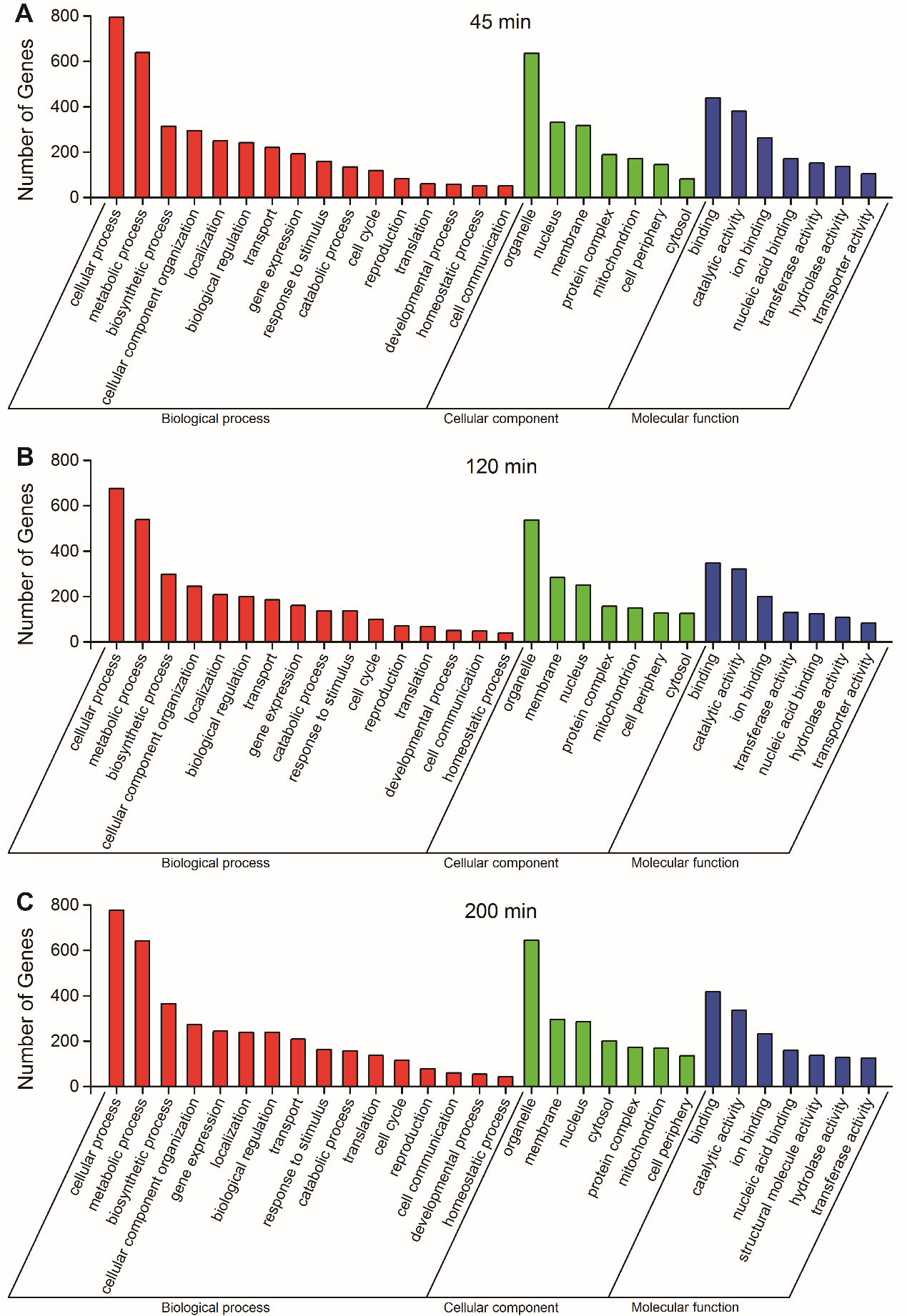


**Fig. S4 KEGG functional classification of DEGs at three time points.**


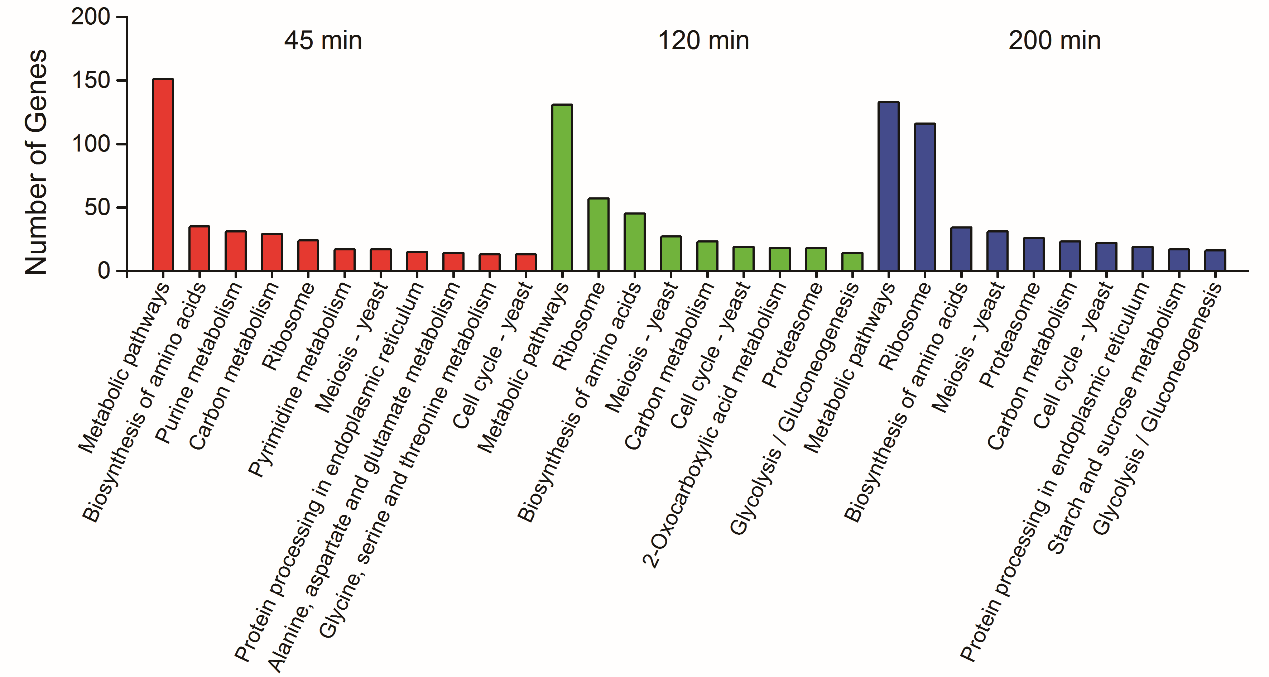


**Fig. S5 Total ion current (TIC) chromatograms of intracellular metabolites in *S. cerevisiae* at three time points.**


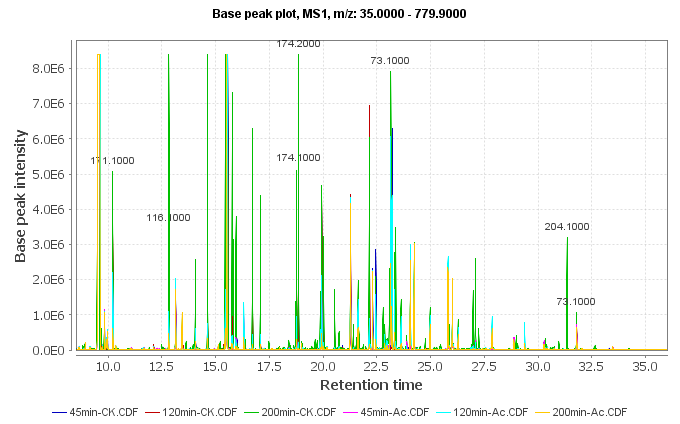


**Fig. S6 PCD assay upon change of histone acetylation balance.** (A) Annexin V/PI costaining of cells overexpressing genes in histone acetylation/deacetylation and vector control. (B, C) Annexin V/PI costaining of mutants and corresponding WT respectively from haploid and diploid strains. (D) DHE staining of acetic acid treated and untreated cells after incubation under different sodium butyrate (SB) concentration.


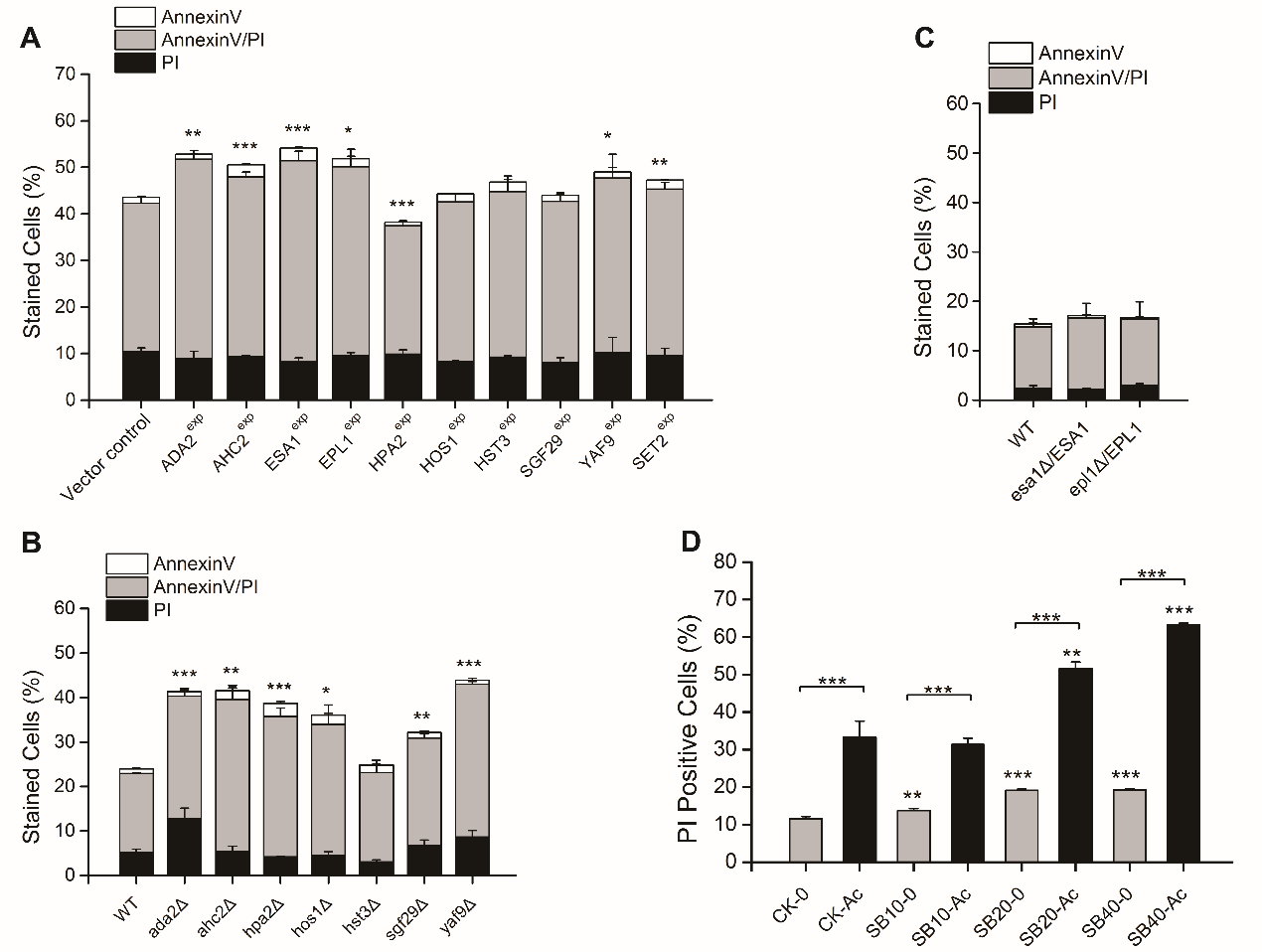

Supplement: Supplementary Tables and Figures [file srep42659-s1.doc]
